# A field vaccine trial in Tanzania demonstrates partial protection against malignant catarrhal fever in cattle

**DOI:** 10.1016/j.vaccine.2015.12.009

**Published:** 2016-02-03

**Authors:** F. Lankester, G.C. Russell, A. Lugelo, A. Ndabigaye, N. Mnyambwa, J. Keyyu, R. Kazwala, D. Grant, A. Percival, D. Deane, D.M. Haig, S. Cleaveland

**Affiliations:** a Boyd Orr Centre for Population and Ecosystem Health, Institute of Biodiversity, Animal Health & Comparative Medicine, University of Glasgow, Glasgow, G12 8QQ, UK; b Paul G. Allen School for Global Animal Health, Washington State University, Pullman, WA 99164, USA; c School of Life Sciences and Bioengineering, Nelson Mandela African Institution of Science & Technology, Arusha, Tanzania; d Moredun Research Institute, Midlothian, Edinburgh, UK; e Faculty of Veterinary Medicine, Sokoine University of Agriculture, Morogoro, Tanzania; f Department of Science and Laboratory Technology, Dar es Salaam Institute of Technology, Dar es Salaam, Tanzania; g Tanzanian Wildlife Research Institute, Arusha, Tanzania; h School of Veterinary Medicine and Science, University of Nottingham, Nottingham, UK

**Keywords:** Malignant catarrhal fever, Alcelaphine herpesvirus 1, Wildebeest, Vaccine field trial, Vaccine efficacy, Tanzania

## Abstract

Malignant catarrhal fever (MCF) is a fatal lymphoproliferative disease of cattle that, in East Africa, results from transmission of the causative virus, alcelaphine herpesvirus 1 (AlHV-1), from wildebeest. A vaccine field trial involving an attenuated AlHV-1 virus vaccine was performed over two wildebeest calving seasons on the Simanjiro Plain of northern Tanzania. Each of the two phases of the field trial consisted of groups of 50 vaccinated and unvaccinated cattle, which were subsequently exposed to AlHV-1 challenge by herding toward wildebeest. Vaccination resulted in the induction of virus-specific and virus-neutralizing antibodies. Some cattle in the unvaccinated groups also developed virus-specific antibody responses but only after the start of the challenge phase of the trial. PCR of DNA from blood samples detected AlHV-1 infection in both groups of cattle but the frequency of infection was significantly lower in the vaccinated groups. Some infected animals showed clinical signs suggestive of MCF but few animals went on to develop fatal MCF, with similar numbers in vaccinated and unvaccinated groups. This study demonstrated a baseline level of MCF-seropositivity among cattle in northern Tanzania of 1% and showed that AlHV-1 virus-neutralizing antibodies could be induced in Tanzanian zebu shorthorn cross cattle by our attenuated vaccine, a correlate of protection in previous experimental trials. The vaccine reduced infection rates by 56% in cattle exposed to wildebeest but protection from fatal MCF could not be determined due to the low number of fatal cases.

## Highlights

• We carried out the first field trial of a vaccine against malignant catarrhal fever.
• The vaccine was shown to be safe for use in Tanzanian cattle.
• The vaccine provided a 56% reduction in transmission of virus to cattle.
• Non-fatal MCF infections in cattle were more common than previously reported.

## 1 Introduction

Malignant catarrhal fever (MCF) is an infectious systemic disease of artiodactyls that is caused by γ-herpesviruses of the genus *Macavirus*. The disease occurs following cross-species transmission from a carrier host that harbours the virus subclinically [30]. Two major epidemiological forms of MCF exist, defined by the reservoir species from which the causative γ-herpesvirus arises: i) wildebeest-associated (WA-MCF) [22] and ii) sheep-associated (SA-MCF) [26]. WA-MCF occurs primarily in sub-Saharan Africa wherever wildebeest come into contact with cattle. The causative pathogen in WA-MCF, alcelaphine herpesvirus-1 (AlHV-1) [22], [27], is excreted principally by wildebeest calves (*Connochaetes taurinus*) in the three months following the brief annual calving period. To avoid disease, pastoralists move their cattle from wildebeest calving grounds, often to more marginal land tens of km away, at a time of year when the condition of cattle is most vulnerable. Consequently, the economic costs associated with MCF in high-risk areas can be significant [3], [4], [12]. SA-MCF, caused by ovine herpesvirus-2 (OvHV-2), occurs worldwide wherever sheep and disease-susceptible animals are in proximity [26]. OvHV-2 is phylogenetically related to AlHV-1 with significant DNA sequence identity [9].

Typical clinical signs of WA-MCF or SA-MCF in cattle include fever, lesions in the oral and nasal mucosa, mucopurulent nasal discharge, corneal opacities and, frequently, death [18], [30]. Histologically, MCF is characterized by vasculitis, epithelial damage and lymphocytic infiltration of tissues [21]. It is currently believed that virus-infected T-cells are responsible for MCF pathogenesis, although the specific mechanisms are not fully resolved [5], [32].

Transmission from reservoir hosts to MCF susceptible animals is thought to be by aerosol transmission and contact with the virus on pasture. For AlHV-1 MCF, there is recent evidence that wildebeest placentae contain virus [13], but its role in the epidemiology of MCF is not clear [4], [29].

There have been several attempts to develop an effective vaccine against MCF [7], [23]. Recent success with an attenuated AlHV-1 vaccine that protected British Holstein-Friesian cattle from experimental intra-nasal challenge with AlHV-1 (mimicking a natural route of transmission) [8], [17], [31] was based on the induction of a mucosal barrier of virus-neutralizing antibodies in the oro-nasal pharyngeal region [8], [31]. This vaccine was effective for six months, which should protect cattle during the wildebeest calving season [31]. In this study, the vaccine was tested for the first time under field trial conditions in northern Tanzania. The trial was timed to coincide with two wildebeest calving seasons (mid-February), with wildebeest calves expected to shed AlHV-1 virus until approximately 3 months of age [20].

## 2 Materials and methods

### 2.1 Study site and baseline serological survey

**Fig. 1:**
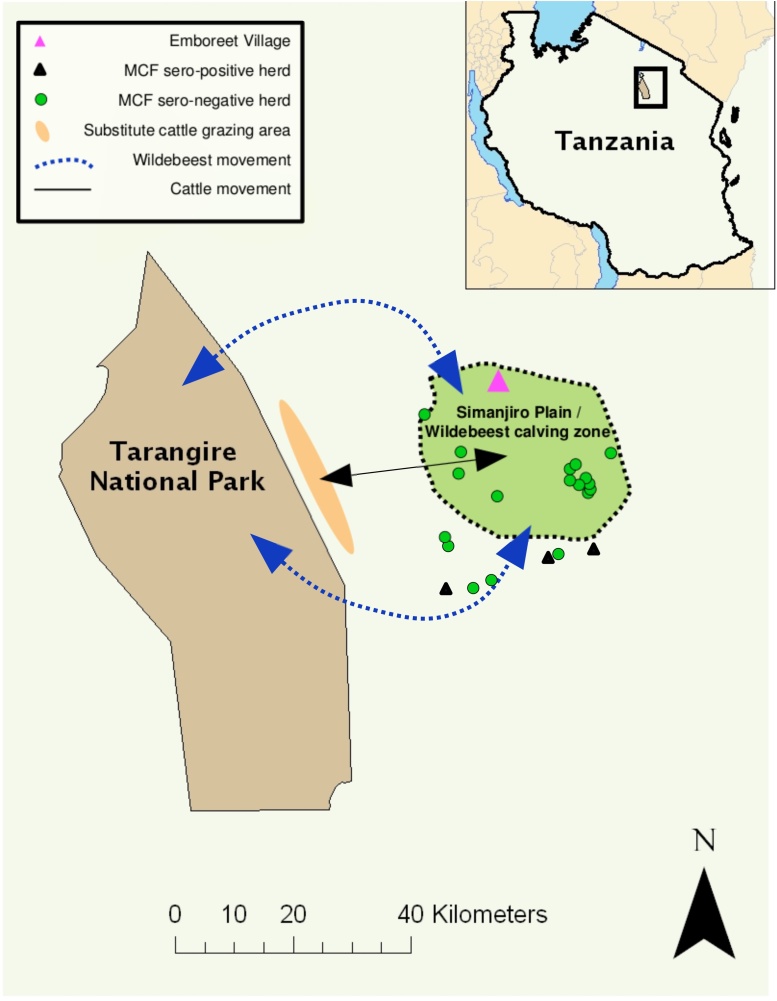
A map indicating the location of the study: Tarangire National Park, the Simanjiro Plain, the wildebeest migration routes (blue dotted line) and the direction that cattle are traditionally herded (black solid line) to find substitute grazing pastures (orange area) are indicated. The locations of the herds that tested MCF seropositive (black triangles) and seronegative (green circles) in the baseline serological screen are also shown.

The study site was the Simanjiro Wildlife Dispersal Area (Simanjiro Plain), a mixed-use livestock grazing and wildlife dispersal area 40 km east of Tarangire National Park in northern Tanzania (latitude -3.952239, longitude 36.47537) (Fig. 1). Prior to the start of the trial, MCF seroprevalence was estimated in cattle herds around the study site. Herds and cattle were selected at random, with a total of 362 cattle sampled from 22 herds in four villages within 40 km of the national park (Fig. 1). Serum samples were heat-treated (56 °C, 30 min) to inactivate adventitious pathogens before being shipped frozen to the Moredun Research Institute, UK, for serological analysis, as described below.

### 2.2 Animals

A total of 200 crossbred Tanzanian shorthorn zebu cattle of approximately six months of age were purchased at the local Sukuro primary livestock market. On arrival at the study site (at least one month before MCF vaccination), all were fitted with ear tags, immunized against East Coast fever (ECF) [6], [10], and treated for endo- and ectoparasites (detailed in Supplementary Data 1).

### 2.3 Field trial design

The field trial was a blinded randomized controlled trial. Sample size calculations indicated that groups of 43 cattle would enable, with 95% confidence and a power of 80%, the detection of a decline in the proportion of exposed animals succumbing to MCF from 30 to 5% [1], [19]. To increase the power, groups of 50 vaccinated and 50 unvaccinated animals were used and the trial was carried out twice, firstly between December 2010 and July 2011 (2011 trial) and secondly between the same months the following year (2012 trial). In each trial, the cattle were randomly divided into a vaccinated group that received a prime and, four weeks later, a boost containing the attenuated AlHV-1 C500 virus mixed with the adjuvant Emulsigen^®^ (20% v/v) administered intramuscularly in the upper neck region, and an unvaccinated group that received a mock prime and boost using a virus-free Emulsigen^®^ inoculum, as described previously [31].

In each trial, the challenge phase began in mid-February and lasted until the end of May. During this period, cattle were grazed as a single herd close to wildebeest and their calves. To estimate the daily intensity of challenge, a contact index was calculated as described in Supplementary Data 2.

Clinical signs of disease (ocular/nasal discharge or lesions; changes in demeanour or appetite) were recorded daily while rectal body temperatures were recorded every second day, or daily if sick. Cattle were classified daily as ‘healthy’, or, if they had temperature ≥39 °C or clinical signs as described above, ‘sick’.

Uncoagulated blood and nasal secretion (NS) samples were collected from all cattle starting at the time of primary inoculation (month zero) and ending six months later. NS samples were collected using a tampon (Lil-lets^®^, regular) inserted into one nostril for 10 min. Plasma and buffy coat cells from uncoagulated blood and NS samples were stored at -20 °C. Prior to exportation to the UK cell-free samples were heat-treated at 56 °C for 30 min.

### 2.4 Pathology and histopathology

In fatal cases, a post-mortem examination was performed and tissue samples (kidney, liver, lung and lymph node) collected and fixed in 10% formalin. Following export to the Moredun Research Institute (licence: POAO(S)/2011/54), the fixed samples were embedded in paraffin wax and stained with hematoxylin and eosin. Histopathological examination enabled the pathology of each case to be summarized as: Category A: MCF (most organs contained significant numbers of lesions consistent with a diagnosis of MCF) [8]; Category B: non-specific infection, consistent with MCF (small number of lesions of mild intensity, without extensive infiltration of lymphocytes or clear vasculitis, in one or two studied organs); or Category C: negative (no significant lesions observed).

### 2.5 Antibody responses

Plasma and NS antibody responses were measured by AlHV-1 virus-specific (ELISA) and virus-neutralizing antibody (VNA) assays, following previously described protocols [8], [31]. MCF-affected cattle can develop virus-specific but not virus-neutralizing antibodies, while induction of neutralizing antibodies in plasma and NS is associated with vaccine-induced protection [8], [31].

ELISA values for each sample were calculated as the difference between means of positive and negative antigen wells for each sample dilution. In the baseline serosurvey, positive samples were defined as those having an ELISA value greater than the cut-off value (mean plus 3 × standard deviation of all samples). In the vaccine field trial, ELISA values were used to calculate a relative titre for each test sample, with respect to standard curves of pooled MCF-positive plasma or NS, with dilutions of 1/20 to 1/6400. ELISA titre values have been expressed as the reciprocal of the calculated titre (e.g. 20−6400). Any sample that gave a calculated titre of less than 20 (i.e. below the range of the standard curve) was not considered positive. VNA analyses were conducted on selected trial cattle at the time of primary vaccination and at the two-month time point, as detailed in Supplementary Data 3. Vaccinated cattle were selected for VNA analysis at random, while all unvaccinated cattle with positive ELISA titres were tested.

### 2.6 Detection of viral DNA in blood

Viral DNA was extracted from frozen buffy coat samples using the ZR Viral DNA Kit™(Zymo Research Coporation, USA) according to the manufacturer's instructions and was assayed by nested PCR as described previously [31]. Animals were classified as AlHV-1 positive if one or more PCR analyses during the challenge phase were positive.

### 2.7 Case definitions

Three case definitions were used for this trial based on PCR detection of AlHV-1 DNA, histopathology and clinical signs:

I. Not infected: AlHV-1 DNA was not detected by PCR in any buffy-coat sample taken during the challenge phase of the trial.
II. AlHV-1 infected: AlHV-1 DNA was detected by PCR and the animal survived or, if the animal died, there were no histopathological lesions indicative of MCF.
III. Fatal MCF: the animal died following clinical signs typical of MCF. AlHV-1 DNA was detected by PCR and post-mortem histopathological findings were consistent with MCF (categories A and B).

### 2.8 Statistical analyses

All statistical analyses were performed using the R language for statistical computing [33]. Linear regression models were used to assess vaccine safety and to determine whether the results were consistent between the two phases of the trial. A Pearson correlation coefficient was used to examine the relationship between virus-specific and -neutralizing antibody titres. Binomial generalized linear models (GLM) were used to analyse the predictive effect of (i) antibody titres on survival and (ii) vaccination status and antibody titres on AlHV-1 infection status.

## 3 Results

### 3.1 Baseline seroprevalence survey

Of the 362 cattle sampled during the baseline survey, four cattle (from three separate herds) were seropositive for MCF, giving an apparent seroprevalence of 1%. The locations of the tested and MCF seropositive herds are shown in Fig. 1.

### 3.2 Vaccine safety

In the 2011 trial, cattle were monitored closely during the two weeks following primary vaccination for signs of ill health. During this two-week period 7% of cattle were recorded as ‘sick’, with the percentage the same in the vaccinated and unvaccinated groups. As regression analysis indicated that vaccination status was not a predictor of daily body temperature during this period (*p* = 0.93, *F* = 0.008, df = 698), the mild sickness was considered unrelated to vaccination. Further, there were no adverse sub-cutaneous reactions at the site of inoculation. We conclude that the vaccine is safe for use in Tanzanian shorthorn zebu cattle.

### 3.3 Contact between wildebeest calves and trial cattle

**Fig. 2:**
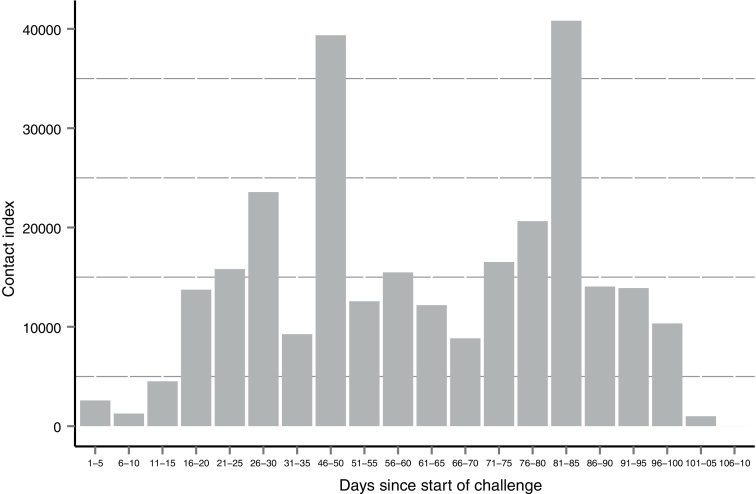
Contact between trial cattle and wildebeest calves. Contact index values (calculated as described in Supplementary Data 2) are plotted in 5-day increments from the start of the challenge phase.

The data used to calculate the daily contact index are shown in Supplementary Data 2 and the temporal contact pattern is shown in Fig. 2. Early in the challenge phase few wildebeest calves were born and the index was low (< 3000). Thereafter, the number of calves in contact increased sharply, to a peak of 142 between day 26 and 30, (contact index > 23,000). Variation in calf numbers appears to drive the pattern of contact up to day 50, after which calf numbers reduce to below 100 and the increase in contact index is driven equally by increasing contact duration and proximity. This might reflect either that the wildebeest were becoming habituated to cattle grazing close by or that the Maasai herders, who had been instructed to graze the trial cattle as close as possible to the wildebeest, were improving their herding skills. Maximum contact index (40,824) occurred between days 81 and 85. The wildebeest began to move away from the Simanjiro Plain around day 100 and the daily contact index decreased to zero by day 106 (Fig. 2).

### 3.4 Field trial outcomes

A complete summary of the individual cattle outcomes from the field trial is shown in Supplementary Data 1.

### 3.5 Clinical, mortality and histopathological data

A summary of the outcomes of the 2011 and 2012 trials is shown in Table 1. Across both trials, three vaccinated and four unvaccinated cattle died after developing clinical signs consistent with MCF. Histopathological examination indicated that all seven cattle had pathology consistent with MCF (Categories A and B) and were PCR positive for AlHV-1 DNA. An additional vaccinated animal died peracutely in the 2012 trial with symptoms typical of black-quarter disease, a bacterial infection most commonly caused by *Clostridium chauvoei* (acute lameness, crepitus and sudden death). There were no lesions observed on histopathology (Category C) but PCR analysis performed on kidney and mediastinal lymph nodes was positive for AlHV-1 DNA.

### 3.6 Analysis of AlHV-1 infection by PCR

**Fig. 3:**
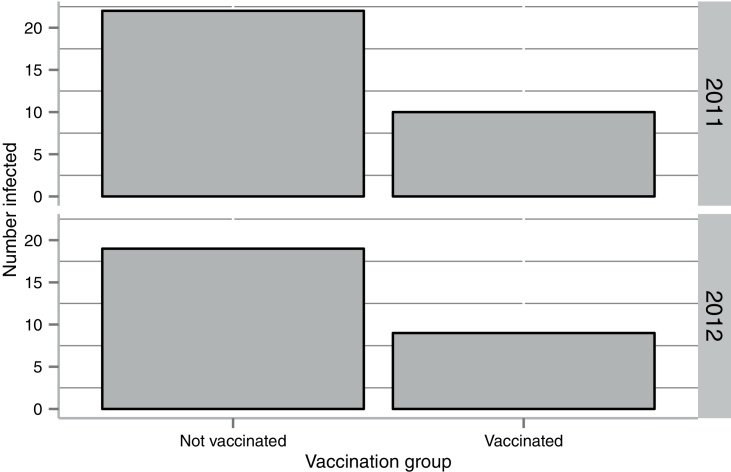
For each of the 2011 and 2012 trials, the number of vaccinated and unvaccinated cattle that tested PCR positive is shown.

AlHV-1 DNA was assayed at three time points during the challenge phase of the trial, following the period of highest contact with wildebeest [21] (see Supplementary Data 2 and 4). Viral DNA was not detected in any sample collected prior to virus challenge. PCR results are summarized by vaccination group in Table 1 and Fig. 3. In total, 45% of unvaccinated cattle became AlHV-1 infected compared with 20% of vaccinated cattle. Modeling indicated that vaccination status was a significant predictor of infection with unvaccinated animals more likely to be infected (*p* = 0.0004, *Z* = -3.5, df = 185).

### 3.7 Case definitions and vaccine efficacy

The clinical, mortality, histopathology and PCR data allowed every animal to be allocated to one of three case definitions summarized in Table 1. The numbers in each category did not differ significantly between the 2011 and 2012 trials (*p* = 0.54, *F* = 0.37, df = 185). In total, 19 of 95 vaccinated cattle became infected with AlHV-1, of which three developed fatal MCF. In the unvaccinated groups, 41 of 92 cattle became infected and four developed fatal MCF. One vaccinated animal was PCR positive for AlHV-1 but died peracutely of suspected black quarter. This was assigned to Case Definition II (AlHV-1 infected) due to the pathognomonic clinical signs of black quarter and the lack of MCF-specific histopathology.

Vaccine efficacy was calculated with respect to preventing AlHV-1 infection (as defined by PCR) according to published methods [11], [15] (Supplementary Data 5). Vaccine efficacy was 56% for preventing infection with AlHV-1. We did not calculate efficacy at preventing fatal MCF as the number of fatal cases in each group was similar.

To investigate the longer-term consequences of non-fatal infections, including possible recrudescence, 27 cattle from the 2011 trial were kept until the end of the 2012 trial. Despite 13 cattle having PCR or serological evidence of non-fatal AlHV-1 infection, none showed any signs of MCF during this extended period.

### 3.8 Serological analysis

**Fig. 4:**
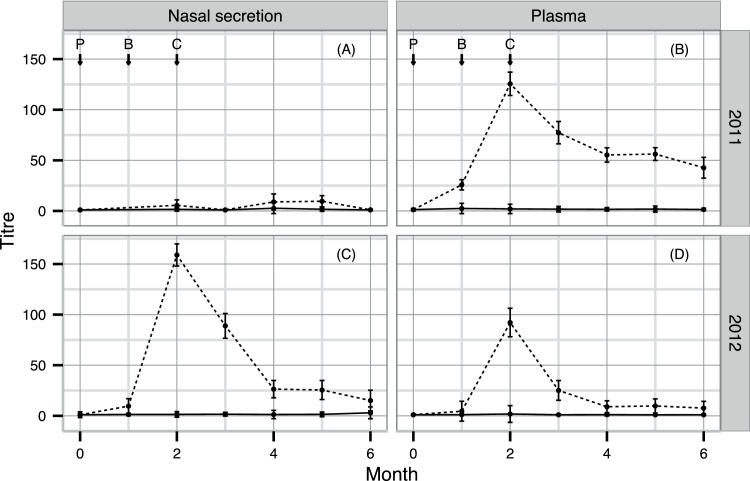
Nasal secretion (plots A & C) and plasma (plots B & D) AlHV-1-specific antibody responses in vaccinated (dashed line) and unvaccinated (solid line) animals from the 2011 and 2012 trials are plotted as geometric means for each month. P = primary vaccination, B = booster vaccination and C = beginning of challenge phase.

The NS and plasma AlHV-1-specific (ELISA) antibody titres for all animals tested at each monthly time point are shown in Supplementary Data 1. The geometric mean NS and plasma ELISA titres for the vaccinated and unvaccinated groups are shown in Fig. 4. All vaccinated animals had positive plasma and NS ELISA titres with mean plasma titres peaking two months after primary immunization. NS ELISA titres also peaked two months after primary immunization in the 2012 trial but, unlike the plasma titres, showed a significantly lower response in the 2011 trial (*p* < 2.2 × 10^−16^, *F* = 173.2, df = 98). During the challenge phase, 40 of the 100 unvaccinated animals had at least one plasma or NS sample return a seropositive result suggestive of exposure to AlHV-1. Despite this, the mean plasma and NS ELISA titres of unvaccinated animals were essentially zero throughout both the 2011 and 2012 trials.

The full data set from the plasma and NS VNA analysis is shown in Supplementary Data 1. All available samples from vaccinated cattle (94) were tested for NS VNA at the point of peak ELISA response, while a subset of these (23) was also tested for plasma VNA at the same point. All unvaccinated cattle with MCF-specific ELISA titre >20 were tested for VNA in both NS and plasma. At peak serological response, 31 and 76% of vaccinated cattle from the 2011 and 2012 trials, respectively, had positive NS VNA titres. No unvaccinated cattle in either trial produced VNA. The geometric mean NS VNA titres of vaccinated cattle in the 2011 trial were significantly lower than the 2012 trial titres (*p* = 3.5 × 10^-5^, *F* = 18.9, df = 92).

### 3.9 The effect of antibody titre on infection status

**Fig. 5:**
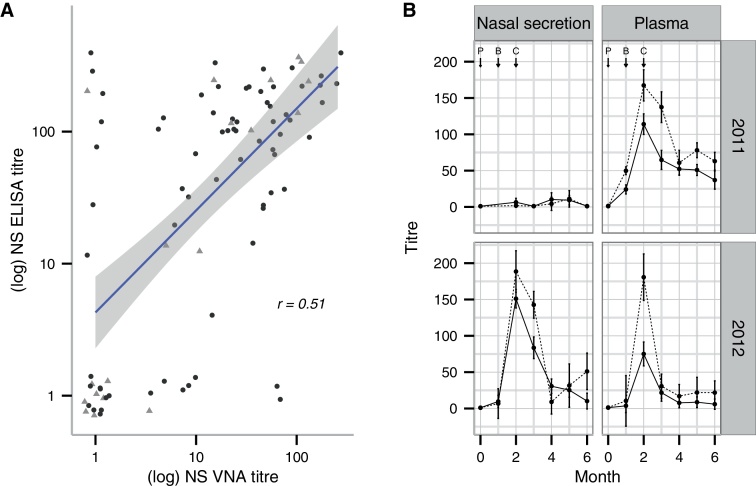
Analysis of serological responses. (A) A correlation of nasal secretion AlHV-1 ELISA and VNA titres (log_10_) of vaccinated cattle only. The line and the shaded area indicate the regression line and associated 95% confidence interval (*r* = correlation coefficient). Subsequent infection status is shown as follows: grey triangles = PCR positive cases; black circles = PCR negative cases. (B) Nasal secretion and plasma geometric mean ELISA titres from vaccinated cattle are plotted for each month during the 2011 and 2012 trials. Dashed lines indicate infected (*n* = 19) and solid lines uninfected cattle (*n* = 76). P = primary vaccination, B = booster vaccination and C = beginning of challenge phase.

At peak serological response, ELISA and VNA titres in vaccinated cattle were significantly correlated in plasma (*p* < 0.02, *r* = 0.25, *t* = 2.4, df = 92) and NS (*p* < 1.0 × 10^−6^, *r* = 0.51, *t* = 5.6, df = 92). Fig. 5A illustrates the correlation and how the titres of infected and uninfected cattle were distributed. Although vaccination status, and thus presence of ELISA and VNA antibodies, has been shown (above) to be a strong predictor of infection status as determined by PCR, modeling indicated that the *magnitude* of ELISA and VNA titres did not have a significant influence on whether vaccinated cattle became infected (*p* > 0.3; Fig. 5B). However the presence of VNA antibodies in NS of vaccinated cattle was weakly associated with protection from infection in vaccinated cattle (*p* = 0.07).

### 3.10 Infection status determined by post-challenge seroconversion

In unvaccinated cattle seroconversion is an indicator of AlHV-1 infection. This can be used in combination with PCR results to determine the proportion of unvaccinated cattle that became infected during challenge (Supplementary Data 1). Of 97 initially seronegative unvaccinated cattle, 40 seroconverted during the challenge phase, while a further 26 remained seronegative but were PCR positive (Table 1). In total, 66 out of 97 (68%) unvaccinated cattle showed evidence of post-challenge infection, of which four died of MCF. The proportion of infected unvaccinated cattle that died of MCF was 6%.

## 4 Discussion

This paper describes the first field trial of a vaccine for wildebeest-associated malignant catarrhal fever, previously tested using experimentally challenged British Holstein-Friesian cattle [8], [17], [31]. The clinical data from this trial indicated that the attenuated virus vaccine formulation was well tolerated, with no evidence of adverse effects. Importantly, vaccinated cattle were more than twice as likely as unvaccinated cattle to remain uninfected with MCF virus transmitted naturally from wildebeest. Due to the unexpectedly low number of fatal MCF cases, we cannot draw conclusions regarding the vaccine's efficacy at reducing lethal MCF. As we are not aware of any herpesvirus vaccines that prevent infection and the establishment of latency, we consider the proportion of vaccinated animals, in both laboratory trials (70 [8] and 81% [31]) and this field trial (80%), that did not become infected in the face of intense challenge as a promising indication for vaccine improvement strategies. Initial work to improve the response induced by the current vaccine strategy by the inclusion of specific toll-like receptor agonists with the adjuvant has been tested in other vaccine trials [17], [38]. However, any further improvement of this vaccine should be done in the context of a natural challenge system that will demonstrate the benefits for livestock keepers.

The serological analysis indicated that the vaccine was effective at eliciting a virus-specific immune response in all vaccinated cattle. ELISA titres peaked two months after primary inoculation. The NS results, however, varied between the trials, with both ELISA and VNA titres being significantly lower in 2011. As the plasma ELISA titres from both trials were similar (Fig. 4), it is possible that these results were caused by a sample processing issue in the 2011 NS samples rather than a failure of the vaccine itself.

The moderately strong correlation between the ELISA and VNA titres in NS and plasma samples (Fig. 5A) was expected, with all cattle that exhibited high VNA titres also having a high ELISA titre. There were, however, some cattle that, despite having relatively high ELISA titres, had low or zero VNA titres. It is unclear why these cattle failed to produce neutralizing antibodies but host genetic or immunological factors could focus virus-specific immune responses on antigens or epitopes that were non-neutralizing. Indeed, two AlHV-1 capsid proteins, which are unlikely to be neutralizing antigens, are strongly recognized by AlHV-1 vaccinated or infected cattle sera [2]. Analysis of NS VNA data from the vaccinated cattle showed that the correlation between the presence of VNA antibodies and protection from infection approached, but did not achieve, statistical significance (*p* < 0.07). This is illustrated in Fig. 5A, where nine of 60 cattle with VNA titre >10 were infected (grey triangles on the right of the figure). This lack of a significant association may indicate that the induction of mucosal VNA is not as strong a correlate of protection as previously thought and that other aspects of the immune response induced by vaccination may also contribute to protection from MCF. It may also reflect differences in the mode of challenge between this trial and previous trials where the source of virus, the dose and the timing of challenge were all controlled.

WA-MCF is reported to have a ‘case-fatality rate’ of between 96 and 100% [3], [22], [28]. However, these reports may be based on observed progression of clinical MCF cases in the absence of capability to estimate sub-clinical infection rates. The baseline seroprevalence analysis of cattle provided preliminary evidence that non-fatal AlHV-1 infections do occur. Similarly, among the trial cattle, four animals had low ELISA titres in plasma at day zero, although none had detectable AlHV-1 DNA at any time point tested. Two of these cattle were subsequently vaccinated and developed high ELISA and VNA titres, while the other two were unvaccinated and had low positive ELISA titres but no VNA titre (Supplementary Data 1). It is unclear whether this apparent pre-exposure to AlHV-1 influenced the outcome of these cattle to subsequent challenge. PCR and serological evidence indicated that 68% of unvaccinated cattle became infected during the challenge phase of the trial, however only 6% of infected cattle developed fatal MCF. This high frequency of non-fatal infections could reflect the true range of outcomes following AlHV-1 infection. Indeed, in SA-MCF, non-fatal infections have been reported [14], [16].

The East Coast fever vaccination, administered to all trial cattle before the MCF vaccine, may have influenced the post-infection outcomes seen in the trial. The ECF vaccine is thought to induce a cell-mediated immune response that suppresses the proliferation of CD8 T-cells [6], [24]. The cell biology and pathogenesis of MCF are not fully understood, but the associated indiscriminate tissue damage is thought to involve virus-infected CD8 T- cells [5], [9], [32], [34]. Thus, the immuno-modulating effects of the ECF vaccination might provide some protection from fatal MCF pathology among all trial cattle and warrants further study.

This trial also provided further insights into natural AlHV-1 infection. For example, most PCR-positive samples came from a time point close to challenge day 70 in both trials. The estimated incubation period for MCF is about 21 days [21], indicating an infection window close to day 49, which coincides with a peak in wildebeest contact index (Fig. 2). These results show that herding cattle close to wildebeest calves of less than three months of age does expose them to AlHV-1 and underscores the accuracy of the timing of the traditional Maasai disease avoidance strategy.

Conservationists have often been concerned that an effective MCF vaccine may result in large-scale, unsustainable shifts in livestock grazing that could cause environmental damage in the important wildlife dispersal areas adjacent to parks and game reserves. However, the partial (56%) protection provided by this vaccine is probably insufficient for pastoralists to risk changing traditional avoidance strategies to graze cattle in productive lands alongside wildebeest during the calving season. Nonetheless, even partial protection would still be of value to protect animals that cannot be moved, for example, where some of the herd remain at the permanent family *boma* to provide milk for women and children attending school [12], or where land-use changes make traditional disease avoidance strategies difficult [25], [37]. A partially protective vaccine may therefore offer a feasible solution to some of the current land-use challenges and conflicts, providing some protection to valuable livestock where avoidance strategies are not possible, but with less risk of potentially damaging environmental consequences. More widely, the vaccine could also play a role around the world in disease prevention strategies where cattle live in close proximity to zoological gardens housing wildebeest calves [35], [36].

## 5 **Research and ethical clearance**

The research was carried out with the approval of the Tanzanian Wildlife Research Institute (TAWIRI), the Commission for Science and Technology (COSTECH, Tanzania) and the Tanzania Food and Drug Administration (permit nos. 2011-213-ER-2005-141 and 2012-318-ER-2005-141). The vaccination trial, including immunization, sampling, clinical scoring and criteria for euthanasia after onset of MCF [31], followed protocols established during trials at the Moredun Research Institute, UK compliant with Home Office of Great Britain and Northern Ireland ‘Animals (Scientific Procedures) Act 1986′ under project licence PPL 60/3839.

## Figures and Tables

**Table 1:** Group specific (vaccinated and unvaccinated) outcomes of the 2011 and 2012 trials.

Trial	Group	Clinical^a^	Died	Histopathology^b^	PCR^c^		Case definition^d^		Unvaccinated, PCR negative, seroconverted^e^
2011	Vaccinated (*n* = 50)	39	2	Category A: 2	Pos	10	I.	38	
					Neg	38	II.	8	
					NT	2	III.	2	
							ND	2	
2011	Unvaccinated (*n* = 50)	46	2	Category A: 1	Pos	22	I.	24	13
				Category B: 1	Neg	24	II.	20	
					NT	4	III.	2	
							ND	4	
2012	Vaccinated (*n* = 50)	50	2	Category A: 1	Pos	9	I.	38	
				Category C: 1	Neg	38	II.	8	
					NT	3	III.	1	
							ND	3	
2012	Unvaccinated (*n* = 50)	49	2	Category B: 2	Pos	19	I.	27	12
					Neg	27	II.	17	
					NT	4	III.	2	
							ND	4	

a Clinical: Number of cattle that were recorded as being sick during the challenge phase.

b Histopathology: Number of fatal cases whose tissues were categorized as: (A) pathology consistent with MCF; (B) pathology consistent with a non-specific infection and (C) no pathology detected.

c PCR: Pos, AlHV-1 DNA detected at one or more time points; Neg, AlHV-1 DNA not detected in any sample; NT = not tested or ambiguous result.

d Case definition: (I) not infected; (II) AlHV-1 infected; (III), fatal MCF; (ND,) not defined.

e Unvaccinated, PCR negative, seroconverted: Number of unvaccinated cattle that had serological but not PCR evidence of AlHV-1 infection.

## Appendix A upplementary data

Supplementary Data 1 The pre-trial medical treatment administered to the cattle is described, plus three tables containing the complete set of plasma and nasal secretion ELISA and virus neutralizing antibody titres are presented.

Supplementary Data 2 How the wildebeest calf Contact Index was measured and calculated is described, plus a table is presented that shows the wildebeest calf contact data for each five-day period of the 2011 trial.

Supplementary Data 3 The selection of plasma and nasal secretion samples for virus neutralizing antibody (VNA) assay is described.

Supplementary Data 4 The full set of PCR results for the 2011 and 2012 trials is shown.

Supplementary Data 5 The method and data used for the vaccine efficacy calculation are described.
