# Supplementary material for: A field vaccine trial in Tanzania demonstrates partial protection against malignant catarrhal fever in cattle
Source: Vaccine. 2016 Feb 3;34(6):831–8. doi: 10.1016/j.vaccine.2015.12.009 (PMC4742522; doi:10.1016/j.vaccine.2015.12.009)
Supplement: Supplementary Data 1 — The pre-trial medical treatment administered to the cattle is described, plus three tables containing the complete set of plasma and nasal secretion ELISA and virus neutralizing antibody titres are presented. [file mmc1.docx]

**Supplementary Data 1. Vaccine trial data**

**Pre-trial treatment**

On arrival at the study site (at least one month before MCF vaccination), all cattle were immunized against the locally prevalent and often fatal lymphoproliferative cattle disease East Coast Fever (ECF) [21] using the infection and treatment immunization method (ITM; an inoculation of Theileria parva sporozoites plus a long-acting formulation of 30% oxytetracycline [22]). Additionally, all cattle were treated for endo- and ectoparasites using 1 ml / 50 kg body weight ivermectin (Ivomec®, Merial Animal Health, Essex, UK), administered by subcutaneous injection, followed by treatment with the ecto-parasiticide alphacypermethrin (Paranex®, Farmbase Ltd, Dar es salaam, Tanzania), administered at 100 mg / litre every two weeks.

Data table notes

| **column heading** | **notes** |
| --- | --- |
| Trial | Year of challenge phase is shown |
| ID | Animal ear tag number |
| Vacc | Vaccination status, y, vaccinated; n, unvaccinated |
| Clinical | Clinical signs of illness during challenge phase as described in text:  y, sick; n, not sick |
| Died | y = animal euthanized for welfare reasons; n = animal survived |
| Histopath | For cattle that died, histopathology classification as described in text |
| Inf. Status | Detection of AlHV-1 infection by PCR: p = positive; n = negative |
| Plasma | Plasma ELISA titre at the monthly time point specified (0-6) |
| NS | Nasal secretion (NS) ELISA titre at the monthly time point specified (0-6) |
| VNA-Pl | Plasma virus neutralizing antibody (VNA) titre at the monthly time point specified (0 or 2) |
| VNA-NS | NS VNA titre at the monthly time point specified (0 or 2) |

The data are presented in three tables containing plasma ELISA, NS ELISA and VNA titres respectively. The remaining columns are presented in each table for ease of use.

Table 1 Plasma ELISA data

| Trial | **ID** | **Vacc** | **Clinical** | **Died** | **Histopath** | **Inf. Status** | **Plasma 0** | **Plasma 1** | **Plasma 2** | **Plasma 3** | **Plasma 4** | **Plasma 5** | **Plasma 6** |
| --- | --- | --- | --- | --- | --- | --- | --- | --- | --- | --- | --- | --- | --- |
| 2011 | 801 | y | y | n |  | n | 0 | 59 | 150 | 132 | 141 | 0 | 82 |
| 2011 | 802 | y | y | n |  | n | 0 | 37 | 1 | 13 | 9 | 31 | 0 |
| 2011 | 803 | n | y | n |  | n | 0 |  | 0 | 0 |  |  | 0 |
| 2011 | 804 | n | y | n |  | n | 0 | 0 | 0 | 0 | 0 | 0 | 0 |
| 2011 | 805 | n | y | n |  | n | 0 | 24 | 82 | 0 | 7 | 0 | 0 |
| 2011 | 806 | y | n | n |  | n | 0 | 0 | 125 | 10 | 93 | 76 | 0 |
| 2011 | 807 | n | y | n |  | n | 0 |  | 0 | 0 |  |  | 0 |
| 2011 | 808 | n | y | n |  | n | 0 |  | 0 | 0 |  |  | 0 |
| 2011 | 809 | y | y | n |  | n | 0 | 67 | 154 | 95 | 104 | 71 | 24 |
| 2011 | 810 | n | y | n |  | n | 0 |  | 0 | 0 |  |  | 0 |
| 2011 | 811 | y | y | n |  | n | 0 | 37 | 0 | 98 | 102 | 67 | 62 |
| 2011 | 812 | n | y | n |  | p | 0 | 0 | 166 | 0 | 0 | 21 | 0 |
| 2011 | 813 | y | y | y | A | p | 0 |  | 149 | 249 |  |  |  |
| 2011 | 814 | n | y | n |  | p | 0 | 2 | 5 | 0 | 0 | 0 | 0 |
| 2011 | 815 | n | y | n |  | p | 0 | 20 | 73 | 70 | 21 | 48 | 25 |
| 2011 | 816 | y | y | n |  | n | 0 | 47 | 156 | 96 | 95 | 48 | 21 |
| 2011 | 817 | n | y | n |  | n | 0 |  | 0 | 0 |  |  | 0 |
| 2011 | 818 | n | y | n |  | p | 0 |  | 0 | 0 |  |  | 0 |
| 2011 | 819 | y | y | n |  | p | 0 | 52 | 111 | 204 | 123 | 119 | 48 |
| 2011 | 820 | y | y | n |  | n | 0 | 76 | 315 | 233 | 99 | 107 | 189 |
| 2011 | 821 | y | y | n |  | n | 0 | 44 | 46 | 194 | 74 | 41 | 58 |
| 2011 | 822 | y | n | n |  | n | 0 | 73 | 349 | 292 | 135 | 147 | 210 |
| 2011 | 823 | n | y | n |  | n | 0 | 0 | 0 | 0 | 0 | 0 | 0 |
| 2011 | 824 | y | y | y | A | p | 0 |  | 230 |  |  |  |  |
| 2011 | 825 | n | n | n |  | n | 0 |  | 0 | 0 |  |  | 0 |
| 2011 | 826 | y | y | n |  | n | 0 | 102 | 262 | 185 | 28 | 156 | 278 |
| 2011 | 827 | n | y | n |  | n | 0 | 0 | 0 | 0 | 0 | 0 | 0 |
| 2011 | 828 | y | y | n |  | p | 0 | 77 | 260 | 117 | 65 | 60 | 125 |
| 2011 | 829 | y | y | n |  | n | 0 | 11 | 252 | 78 | 43 | 22 | 3 |
| 2011 | 830 | n | y | n |  | p | 3 | 0 | 0 | 0 | 0 | 12 | 0 |
| 2011 | 831 | y | y | n |  | n | 0 | 30 | 152 | 0 | 33 | 36 | 43 |
| 2011 | 832 | n | n | n |  | p | 0 |  | 0 | 0 |  |  | 0 |
| 2011 | 833 | n | y | n |  | n | 0 |  | 0 | 0 |  |  | 0 |
| 2011 | 834 | y | y | n |  | p | 0 | 40 | 247 | 202 | 135 | 97 | 103 |
| 2011 | 835 | n | n | n |  | p | 0 |  | 0 | 0 |  |  | 0 |
| 2011 | 836 | n | y | n |  | p | 0 |  | 0 | 0 |  |  | 0 |
| 2011 | 837 | n | y | n |  | p | 0 |  | 0 | 0 |  |  | 0 |
| 2011 | 838 | n | y | n |  | p | 0 |  | 0 | 0 |  |  | 0 |
| 2011 | 839 | y | y | n |  | p | 0 | 44 | 71 | 167 | 135 | 117 | 39 |
| 2011 | 840 | n | y | n |  | p | 0 |  | 0 | 0 |  |  | 0 |
| 2011 | 841 | y | y | n |  | n | 0 | 41 | 46 | 0 | 55 | 61 | 0 |
| 2011 | 842 | y | y | n |  | p | 0 | 67 | 178 | 126 | 15 | 34 | 77 |
| 2011 | 843 | n | y | n |  | p | 0 |  | 0 | 0 |  |  | 0 |
| 2011 | 844 | y | y | n |  | n | 0 | 89 | 200 | 142 | 90 | 104 | 137 |
| 2011 | 845 | y | n | n |  | n | 0 | 41 | 0 | 0 | 64 | 69 | 51 |
| 2011 | 846 | y | y | n |  | n | 0 | 45 | 209 | 122 | 0 | 32 | 115 |
| 2011 | 847 | y | y | n |  | p | 0 | 57 | 106 | 29 | 44 | 85 | 91 |
| 2011 | 848 | n | y | n |  | p | 0 |  | 0 | 0 |  |  | 0 |
| 2011 | 849 | y | y | n |  | n | 0 | 96 | 294 | 178 | 155 | 124 | 266 |
| 2011 | 850 | y | y | n |  | n | 0 | 0 | 156 | 179 | 135 | 114 | 117 |
| 2011 | 851 | n | y | n |  | p | 0 |  | 0 | 0 |  |  | 0 |
| 2011 | 852 | y | n | n |  | n | 0 | 43 | 192 | 92 | 118 | 93 | 61 |
| 2011 | 853 | y | y | n |  | n | 0 | 67 | 199 | 82 | 137 | 0 | 0 |
| 2011 | 854 | n | y | n |  | p | 0 |  | 0 | 0 |  |  | 0 |
| 2011 | 855 | y | y | n |  | p | 0 | 30 | 237 | 140 | 26 | 67 | 67 |
| 2011 | 856 | n | y | n |  | n | 0 |  | 0 | 0 |  |  | 0 |
| 2011 | 857 | n | y | y | A | p | 0 |  | 0 | 107 |  |  |  |
| 2011 | 858 | n | y | n |  | n | 0 |  | 0 | 0 |  |  | 0 |
| 2011 | 859 | n | y | n |  | p | 0 |  | 0 | 0 |  |  | 0 |
| 2011 | 860 | y | y | n |  | n | 0 | 10 | 145 | 36 | 56 | 81 | 186 |
| 2011 | 861 | y | y | n |  | n | 0 | 0 | 218 | 143 | 8 | 43 | 26 |
| 2011 | 862 | n | y | n |  | n | 0 |  | 0 | 0 |  |  | 0 |
| 2011 | 863 | n | y | n |  | n | 0 |  | 0 | 0 |  |  | 0 |
| 2011 | 864 | y | y | n |  | n | 0 | 157 | 166 | 53 | 50 | 61 | 0 |
| 2011 | 865 | n | y | n |  | p | 0 | 67 | 39 | 16 | 21 | 0 | 0 |
| 2011 | 866 | n | y | n |  | n | 0 | 0 | 0 | 39 | 0 | 0 | 0 |
| 2011 | 867 | y | y | n |  | p | 0 | 47 | 222 | 170 | 75 | 88 | 22 |
| 2011 | 868 | n | y | n |  | n | 0 | 0 | 112 | 0 | 0 | 0 | 0 |
| 2011 | 869 | n | y | y | B | p | 0 |  | 0 |  |  |  |  |
| 2011 | 870 | n | y | n |  |  | 0 |  | 0 | 0 |  |  | 0 |
| 2011 | 871 | y | n | n |  | n | 0 | 58 | 349 | 221 | 70 |  | 101 |
| 2011 | 872 | n | y | n |  | n | 0 |  | 0 | 0 |  |  | 0 |
| 2011 | 873 | n | n | n |  | n | 21 | 0 | 22 | 18 | 0 | 0 | 26 |
| 2011 | 874 | y | n | n |  | n | 41 | 103 | 141 | 175 | 169 | 143 | 124 |
| 2011 | 875 | y | y | n |  | n | 45 | 0 | 222 | 161 | 60 | 41 | 101 |
| 2011 | 876 | y | y | n |  |  | 3 | 0 | 202 | 187 | 134 | 131 | 183 |
| 2011 | 877 | y | y | n |  | n | 16 | 0 | 164 | 113 | 101 | 119 | 107 |
| 2011 | 878 | n | y | n |  | n | 14 | 0 | 8 | 0 | 0 | 0 | 8 |
| 2011 | 879 | n | y | n |  | p | 13 | 57 | 3 | 20 | 0 | 2 | 7 |
| 2011 | 880 | n | y | n |  |  | 6 | 0 | 68 | 13 | 0 | 0 | 15 |
| 2011 | 881 | n | y | n |  |  | 19 | 0 | 1 | 20 | 0 | 0 | 45 |
| 2011 | 882 | y | y | n |  | n | 0 | 20 | 176 | 60 | 16 | 38 | 16 |
| 2011 | 883 | n | y | n |  | n | 0 |  | 0 | 0 |  |  | 0 |
| 2011 | 884 | y | y | n |  | n | 7 | 60 | 273 | 189 | 34 | 59 | 130 |
| 2011 | 885 | y | y | n |  | n | 0 | 94 | 227 | 267 | 137 | 84 | 195 |
| 2011 | 886 | y | n | n |  | n | 0 | 69 | 196 | 198 | 105 | 116 | 116 |
| 2011 | 887 | y | y | n |  | n | 0 | 122 | 195 | 261 | 141 | 141 | 176 |
| 2011 | 888 | y | n | n |  | n | 0 | 24 | 225 | 146 | 135 | 109 | 112 |
| 2011 | 889 | y | n | n |  | n | 0 | 26 | 172 | 111 | 123 | 116 | 115 |
| 2011 | 890 | n | y | n |  |  | 0 |  | 0 | 0 |  |  | 0 |
| 2011 | 891 | y | y | n |  |  | 0 | 42 | 179 | 151 | 85 | 67 | 100 |
| 2011 | 892 | n | n | n |  | n | 0 |  | 0 | 0 |  |  | 0 |
| 2011 | 893 | y | n | n |  | n | 0 | 6 | 75 | 0 | 2 | 0 | 0 |
| 2011 | 894 | n | y | n |  | n | 0 | 0 | 0 | 0 | 0 | 0 | 0 |
| 2011 | 895 | y | n | n |  | n | 0 | 0 | 106 | 6 | 0 | 18 | 0 |
| 2011 | 896 | n | y | n |  | p | 0 |  | 0 | 0 |  |  | 0 |
| 2011 | 897 | n | y | n |  | p | 0 |  | 0 | 0 |  |  | 0 |
| 2011 | 898 | n | y | n |  | n | 0 |  | 0 | 0 |  |  | 0 |
| 2011 | 899 | y | y | n |  | n | 0 | 31 | 158 | 148 | 130 | 107 | 133 |
| 2011 | 900 | y | y | n |  | n | 0 | 9 | 98 | 129 |  | 161 | 125 |
| 2012 | 1 | y | y | n |  | n | 0 | 0 | 104 | 37 | 0 | 82 | 48 |
| 2012 | 2 | n | y | n |  | n | 0 | 0 | 0 | 0 | 0 | 0 | 0 |
| 2012 | 3 | n | y | y | B | p | 0 | 0 | 0 | 0 | 0 | 0 |  |
| 2012 | 4 | y | y | n |  | p | 0 | 2 | 314 | 86 | 51 | 125 | 94 |
| 2012 | 5 | y | y | n |  | n | 0 | 56 | 289 | 201 | 93 | 56 | 104 |
| 2012 | 6 | y | y | n |  | p | 0 | 317 | 345 | 133 | 141 | 93 | 45 |
| 2012 | 7 | n | y | n |  | n | 0 | 0 | 0 | 0 | 0 | 0 | 0 |
| 2012 | 8 | n | y | n |  | n | 0 | 0 | 0 | 0 | 0 | 0 | 0 |
| 2012 | 9 | n | y | n |  | p | 0 | 0 | 0 | 0 | 0 | 0 | 0 |
| 2012 | 10 | n | y | n |  |  | 0 | 0 | 0 | 0 | 0 | 0 | 0 |
| 2012 | 11 | y | y | n |  | n | 0 | 0 | 88 | 0 | 0 | 20 | 0 |
| 2012 | 12 | y | y | y | A | p | 0 | 0 | 252 | 10 | 0 | 83 |  |
| 2012 | 13 | y | y | n |  | n | 0 | 0 | 58 | 105 | 0 | 0 | 0 |
| 2012 | 14 | y | y | n |  | n | 0 | 55 | 320 | 0 | 14 | 65 | 0 |
| 2012 | 15 | n | y | n |  | n | 0 | 19 | 0 | 0 | 0 | 0 | 0 |
| 2012 | 16 | y | y | n |  | n | 0 | 63 | 212 | 68 | 67 | 30 | 46 |
| 2012 | 17 | n | y | n |  | n | 0 | 0 | 0 | 0 | 0 | 0 | 0 |
| 2012 | 18 | y | y | n |  | p | 12 | 0 | 215 | 43 | 37 | 62 | 0 |
| 2012 | 19 | y | y | n |  | n | 0 | 102 | 113 | 0 | 18 | 54 | 0 |
| 2012 | 20 | n | y | n |  | n | 0 | 0 | 0 | 0 | 0 | 0 | 0 |
| 2012 | 21 | y | y | n |  | n | 0 | 0 | 9 | 0 | 24 | 60 | 86 |
| 2012 | 22 | y | y | n |  | n | 0 | 71 | 152 | 60 | 107 | 91 | 0 |
| 2012 | 23 | y | y | n |  | n | 0 | 34 | 213 | 0 | 74 | 81 | 0 |
| 2012 | 24 | n | y | n |  | n | 0 | 16 | 0 | 0 | 0 | 0 | 0 |
| 2012 | 25 | n | y | n |  | p | 0 | 0 | 0 | 0 | 0 | 0 | 0 |
| 2012 | 26 | y | y | n |  | n | 0 | 0 | 0 | 0 | 0 | 0 | 0 |
| 2012 | 27 | y | y | n |  | n | 0 | 20 | 229 | 90 | 82 | 122 | 16 |
| 2012 | 28 | y | y | n |  | p | 0 | 102 | 65 | 0 | 0 | 0 | 0 |
| 2012 | 29 | n | y | n |  | n | 0 | 0 | 0 | 0 | 0 | 0 | 0 |
| 2012 | 30 | n | y | n |  | n | 0 | 0 | 0 | 0 | 0 | 0 | 0 |
| 2012 | 31 | n | y | n |  | n | 0 | 0 | 0 | 0 | 0 | 0 | 0 |
| 2012 | 32 | n | y | n |  | p | 0 | 0 | 0 | 0 | 0 | 0 | 0 |
| 2012 | 33 | y | y | n |  | n | 0 | 0 | 94 | 0 | 0 | 16 | 0 |
| 2012 | 34 | y | y | n |  | p | 0 | 26 | 172 | 95 | 24 | 153 | 108 |
| 2012 | 35 | n | y | n |  | n | 0 | 0 | 0 | 0 | 0 | 0 | 0 |
| 2012 | 36 | y | y | n |  | n | 0 | 0 | 131 | 76 | 0 | 0 | 0 |
| 2012 | 37 | n | y | n |  | p | 0 | 0 | 0 | 0 | 0 | 0 | 0 |
| 2012 | 38 | n | y | n |  | n | 0 | 0 | 0 | 0 | 0 | 0 | 0 |
| 2012 | 39 | y | y | n |  | n | 0 | 0 | 107 | 56 | 0 | 57 | 0 |
| 2012 | 40 | y | y | n |  | n | 0 | 0 | 67 | 0 | 0 | 0 | 0 |
| 2012 | 41 | n | y | n |  | n | 0 | 0 | 0 | 0 | 0 | 0 | 0 |
| 2012 | 42 | n | y | n |  | n | 0 | 0 | 0 | 0 | 0 | 0 | 0 |
| 2012 | 43 | y | y | n |  | n | 0 | 0 | 222 | 203 | 92 | 169 | 57 |
| 2012 | 44 | y | y | n |  | n | 0 | 0 | 59 | 0 | 0 | 0 | 0 |
| 2012 | 45 | y | y | n |  | n | 0 | 0 | 113 | 44 | 0 | 0 | 0 |
| 2012 | 46 | n | y | n |  |  | 0 | 0 | 0 | 0 | 0 | 0 | 0 |
| 2012 | 47 | n | y | n |  | n | 0 | 0 | 0 | 0 | 0 | 0 | 0 |
| 2012 | 48 | y | y | n |  | n | 15 | 0 | 63 | 127 | 52 | 64 | 28 |
| 2012 | 49 | y | y | n |  | n | 0 | 0 | 250 | 30 | 142 | 0 | 112 |
| 2012 | 50 | y | y | n |  | n | 0 | 0 | 17 | 141 | 34 | 56 | 68 |
| 2012 | 51 | n | y | n |  |  | 0 | 13 | 0 | 0 | 0 | 0 | 0 |
| 2012 | 52 | n | y | n |  | n | 0 | 0 | 66 | 0 | 0 | 0 | 0 |
| 2012 | 53 | n | y | n |  | p | 0 | 0 | 0 | 0 | 0 | 0 | 32 |
| 2012 | 54 | n | y | n |  | p | 0 | 0 | 0 | 0 | 0 | 0 | 0 |
| 2012 | 55 | n | y | n |  | p | 0 | 0 | 226 | 0 | 0 | 0 | 0 |
| 2012 | 56 | y | y | n |  | n | 0 | 0 | 153 | 248 | 94 | 55 | 157 |
| 2012 | 57 | y | y | n |  |  | 0 | 8 | 157 | 119 | 0 | 0 | 30 |
| 2012 | 58 | y | y | n |  | n | 0 | 0 | 0 | 0 | 0 | 54 | 0 |
| 2012 | 59 | n | y | n |  | n | 0 | 0 | 0 | 0 | 0 | 0 | 0 |
| 2012 | 60 | n | y | n |  | p | 0 | 0 | 155 | 0 | 0 | 0 | 0 |
| 2012 | 61 | y | y | n |  | n | 0 | 74 | 0 | 35 | 0 | 0 | 0 |
| 2012 | 62 | n | y | n |  | n | 0 | 0 | 0 | 0 | 0 | 0 | 0 |
| 2012 | 63 | n | y | n |  | n | 0 | 0 | 267 | 0 | 0 | 0 | 0 |
| 2012 | 64 | y | y | n |  | n | 0 | 0 | 45 | 71 | 0 | 150 | 75 |
| 2012 | 65 | y | y | n |  | n | 0 | 0 | 19 | 0 | 0 | 0 | 0 |
| 2012 | 66 | y | y | n |  | p | 0 | 0 | 83 | 21 | 54 | 6 | 75 |
| 2012 | 67 | y | y | n |  | n | 0 | 0 | 0 | 123 | 44 | 0 | 24 |
| 2012 | 68 | n | y | n |  | p | 0 | 0 | 0 | 0 | 0 | 0 | 0 |
| 2012 | 69 | n | y | n |  | p | 0 | 0 | 178 | 0 | 0 | 0 | 0 |
| 2012 | 70 | y | y | n |  | n | 0 | 0 | 259 | 44 | 106 | 21 | 27 |
| 2012 | 71 | y | y | y | C |  | 0 | 0 | 136 |  |  |  |  |
| 2012 | 72 | y | y | n |  | n | 0 | 6 | 157 | 51 | 0 | 0 | 0 |
| 2012 | 73 | n | y | n |  | n | 0 | 0 | 0 | 0 | 0 | 0 | 0 |
| 2012 | 74 | n | y | n |  | p | 0 | 0 | 0 | 0 | 0 | 0 | 0 |
| 2012 | 75 | n | y | n |  | p | 0 | 0 | 0 | 0 | 0 | 0 | 0 |
| 2012 | 76 | n | y | n |  | p | 0 | 0 | 0 | 0 | 0 | 0 | 0 |
| 2012 | 77 | y | y | n |  | n | 0 | 0 | 332 | 189 | 93 | 42 | 107 |
| 2012 | 78 | y | y | n |  |  | 0 | 0 | 155 | 168 | 37 | 120 | 150 |
| 2012 | 79 | y | y | n |  | n | 0 | 0 | 271 | 226 | 57 | 2 | 123 |
| 2012 | 80 | y | y | n |  | n | 0 | 12 | 245 | 95 | 5 | 0 | 8 |
| 2012 | 81 | n | y | y | B | p | 0 | 0 | 0 | 0 | 0 |  |  |
| 2012 | 82 | n | y | n |  |  | 0 | 0 | 0 | 0 | 0 | 0 | 0 |
| 2012 | 83 | y | y | n |  | n | 0 | 170 | 258 | 101 | 0 | 0 | 0 |
| 2012 | 84 | n | y | n |  | n | 0 | 0 | 0 | 0 | 0 | 0 | 0 |
| 2012 | 85 | n | y | n |  | p | 0 | 0 | 0 | 0 | 0 | 0 | 0 |
| 2012 | 86 | n | y | n |  | n | 0 | 0 | 0 | 0 | 0 | 0 | 0 |
| 2012 | 87 | y | y | n |  | p | 0 | 85 | 209 | 80 | 20 | 0 | 68 |
| 2012 | 88 | n | y | n |  | p | 0 | 0 | 0 | 0 | 0 | 0 | 0 |
| 2012 | 89 | y | y | n |  | n | 0 | 269 | 210 | 0 | 0 | 0 | 8 |
| 2012 | 90 | n | y | n |  | n | 0 | 0 | 0 | 0 | 0 | 0 | 0 |
| 2012 | 91 | n | n | n |  | n | 0 | 0 | 0 | 0 | 0 | 0 | 0 |
| 2012 | 92 | y | y | n |  | n | 0 | 0 | 275 | 128 | 42 | 0 | 0 |
| 2012 | 93 | n | y | n |  | n | 31 | 0 | 52 | 32 | 50 | 0 | 0 |
| 2012 | 94 | y | y | n |  |  | 0 | 141 | 247 | 39 | 34 | 0 | 1 |
| 2012 | 95 | n | y | n |  | n | 0 | 0 | 0 | 0 | 36 | 0 | 0 |
| 2012 | 96 | n | y | n |  | n | 0 | 0 | 0 | 0 | 0 | 0 | 0 |
| 2012 | 97 | n | y | n |  | p | 0 |  | 0 | 0 | 0 | 0 | 0 |
| 2012 | 98 | y | y | n |  | n | 0 |  | 201 | 160 | 22 | 0 | 9 |
| 2012 | 99 | n | y | n |  | p | 0 |  | 0 | 0 | 0 | 0 | 0 |
| 2012 | 100 | y | y | n |  | n | 0 |  | 191 | 77 | 0 | 2 | 0 |

Table 2. NS ELISA data

| **Trial** | **ID** | **Vacc** | **Clinical** | **Died** | **Histopath** | **Inf. Status** | **NS 0** | **NS 1** | **NS 2** | **NS 3** | **NS 4** | **NS 5** | **NS 6** |
| --- | --- | --- | --- | --- | --- | --- | --- | --- | --- | --- | --- | --- | --- |
| 2011 | 801 | y | y | n |  | n | 0 |  | 98 | 0 | 183 | 0 | 0 |
| 2011 | 802 | y | y | n |  | n | 0 |  | 0 | 0 | 102 | 0 | 0 |
| 2011 | 803 | n | y | n |  | n | 0 |  | 0 | 0 | 141 | 32 | 0 |
| 2011 | 804 | n | y | n |  | n | 0 |  | 0 | 0 | 0 | 0 | 0 |
| 2011 | 805 | n | y | n |  | n | 0 |  | 0 | 0 | 39 | 0 | 0 |
| 2011 | 806 | y | n | n |  | n | 0 |  | 0 | 0 | 156 | 109 | 0 |
| 2011 | 807 | n | y | n |  | n | 0 |  | 0 | 0 | 31 | 0 | 0 |
| 2011 | 808 | n | y | n |  | n | 0 |  | 0 | 0 | 8 | 0 | 0 |
| 2011 | 809 | y | y | n |  | n | 0 |  | 96 | 0 | 6 | 116 | 0 |
| 2011 | 810 | n | y | n |  | n | 0 |  | 0 | 0 |  | 0 | 0 |
| 2011 | 811 | y | y | n |  | n | 0 |  | 0 | 0 | 0 | 119 | 0 |
| 2011 | 812 | n | y | n |  | p | 0 |  | 0 | 0 | 0 | 0 | 0 |
| 2011 | 813 | y | y | y | A | p | 0 |  | 0 | 0 |  |  | 0 |
| 2011 | 814 | n | y | n |  | p | 0 |  | 0 | 0 | 0 | 0 | 0 |
| 2011 | 815 | n | y | n |  | p | 0 |  | 0 | 0 | 0 | 0 | 0 |
| 2011 | 816 | y | y | n |  | n | 0 |  | 0 | 0 | 90 | 16 | 0 |
| 2011 | 817 | n | y | n |  | n | 0 |  | 26 | 0 | 44 | 0 | 0 |
| 2011 | 818 | n | y | n |  | p | 0 |  | 0 | 0 | 166 | 0 | 0 |
| 2011 | 819 | y | y | n |  | p | 0 |  | 0 | 0 | 53 | 29 | 0 |
| 2011 | 820 | y | y | n |  | n | 0 |  | 37 | 0 | 0 | 0 | 24 |
| 2011 | 821 | y | y | n |  | n | 0 |  | 0 | 0 | 36 | 0 | 0 |
| 2011 | 822 | y | n | n |  | n | 0 |  | 0 | 0 | 0 | 46 | 0 |
| 2011 | 823 | n | y | n |  | n | 0 |  | 0 | 0 | 0 | 0 | 0 |
| 2011 | 824 | y | y | y | A | p | 0 |  | 0 | 0 |  |  | 0 |
| 2011 | 825 | n | n | n |  | n | 0 |  | 0 | 0 | 0 | 0 | 0 |
| 2011 | 826 | y | y | n |  | n | 0 |  | 91 | 0 | 198 | 130 | 0 |
| 2011 | 827 | n | y | n |  | n | 0 |  | 0 | 0 |  | 0 | 0 |
| 2011 | 828 | y | y | n |  | p | 0 |  | 0 | 0 | 0 | 0 | 0 |
| 2011 | 829 | y | y | n |  | n | 0 |  | 0 | 0 | 0 | 38 | 0 |
| 2011 | 830 | n | y | n |  | p | 0 |  | 0 | 0 | 0 | 0 | 0 |
| 2011 | 831 | y | y | n |  | n | 0 |  | 5 | 0 | 0 | 39 | 5 |
| 2011 | 832 | n | n | n |  | p | 0 |  | 0 | 0 | 0 | 0 | 0 |
| 2011 | 833 | n | y | n |  | n | 0 |  | 0 | 0 | 0 | 0 | 0 |
| 2011 | 834 | y | y | n |  | p | 0 |  | 19 | 6 | 0 | 64 | 0 |
| 2011 | 835 | n | n | n |  | p | 0 |  | 0 | 0 | 0 | 0 | 0 |
| 2011 | 836 | n | y | n |  | p | 0 |  | 0 | 0 |  | 55 | 0 |
| 2011 | 837 | n | y | n |  | p | 0 |  | 0 | 0 | 0 | 0 | 0 |
| 2011 | 838 | n | y | n |  | p | 0 |  | 0 | 0 | 0 | 0 | 0 |
| 2011 | 839 | y | y | n |  | p | 0 |  | 0 | 0 | 0 | 72 | 0 |
| 2011 | 840 | n | y | n |  | p | 0 |  | 0 | 0 | 0 | 0 | 0 |
| 2011 | 841 | y | y | n |  | n | 0 |  | 0 | 0 | 0 | 0 | 0 |
| 2011 | 842 | y | y | n |  | p | 0 |  | 0 | 0 | 45 | 26 | 0 |
| 2011 | 843 | n | y | n |  | p | 0 |  | 0 | 0 | 0 | 0 | 0 |
| 2011 | 844 | y | y | n |  | n | 0 |  | 0 | 0 | 39 | 63 | 0 |
| 2011 | 845 | y | n | n |  | n | 0 |  | 0 | 0 | 0 | 0 | 0 |
| 2011 | 846 | y | y | n |  | n | 2 |  | 89 | 0 | 15 | 28 | 0 |
| 2011 | 847 | y | y | n |  | p | 0 |  | 17 | 0 | 0 | 0 | 0 |
| 2011 | 848 | n | y | n |  | p | 0 |  | 0 | 0 | 0 | 0 | 0 |
| 2011 | 849 | y | y | n |  | n | 0 |  | 26 | 0 | 0 | 29 | 0 |
| 2011 | 850 | y | y | n |  | n | 0 |  | 48 | 0 | 54 | 47 | 0 |
| 2011 | 851 | n | y | n |  | p | 0 |  | 0 | 0 | 0 | 0 | 0 |
| 2011 | 852 | y | n | n |  | n | 0 |  | 52 | 0 | 79 | 53 | 0 |
| 2011 | 853 | y | y | n |  | n | 0 |  | 19 | 0 | 32 | 39 | 0 |
| 2011 | 854 | n | y | n |  | p | 0 |  | 0 | 0 | 65 | 0 | 0 |
| 2011 | 855 | y | y | n |  | p | 0 |  | 1 | 0 | 52 | 0 | 0 |
| 2011 | 856 | n | y | n |  | n | 0 |  | 0 | 0 | 0 | 62 | 0 |
| 2011 | 857 | n | y | y | A | p | 0 |  | 1 | 0 |  |  | 0 |
| 2011 | 858 | n | y | n |  | n | 0 |  | 0 | 0 | 0 | 0 | 0 |
| 2011 | 859 | n | y | n |  | p | 0 |  | 5 | 0 | 0 | 0 | 0 |
| 2011 | 860 | y | y | n |  | n | 0 |  | 0 | 0 | 2 | 0 | 0 |
| 2011 | 861 | y | y | n |  | n | 0 |  | 1 | 0 | 16 | 57 | 0 |
| 2011 | 862 | n | y | n |  | n | 0 |  | 0 | 0 | 0 | 0 | 0 |
| 2011 | 863 | n | y | n |  | n | 0 |  | 0 | 0 | 10 | 0 | 0 |
| 2011 | 864 | y | y | n |  | n | 0 |  | 29 | 0 | 0 | 90 | 0 |
| 2011 | 865 | n | y | n |  | p | 0 |  | 59 | 0 | 0 | 22 | 0 |
| 2011 | 866 | n | y | n |  | n | 0 |  | 6 | 0 | 0 | 42 | 0 |
| 2011 | 867 | y | y | n |  | p | 0 |  | 0 | 0 | 0 | 71 | 0 |
| 2011 | 868 | n | y | n |  | n | 0 |  | 0 | 0 | 0 | 0 | 0 |
| 2011 | 869 | n | y | y | B | p | 0 |  | 22 | 0 |  |  | 0 |
| 2011 | 870 | n | y | n |  |  | 0 |  | 0 | 0 | 44 | 0 | 0 |
| 2011 | 871 | y | n | n |  | n | 0 |  | 79 | 0 | 59 | 60 | 0 |
| 2011 | 872 | n | y | n |  | n | 0 |  | 14 | 0 | 37 | 17 | 0 |
| 2011 | 873 | n | n | n |  | n | 0 |  | 61 | 0 | 0 | 0 | 0 |
| 2011 | 874 | y | n | n |  | n | 0 |  | 48 | 0 | 0 | 37 | 0 |
| 2011 | 875 | y | y | n |  | n | 0 |  | 78 | 0 | 53 | 7 | 0 |
| 2011 | 876 | y | y | n |  |  | 0 |  | 181 | 0 | 128 | 60 | 0 |
| 2011 | 877 | y | y | n |  | n | 0 |  | 0 | 0 | 41 | 0 | 0 |
| 2011 | 878 | n | y | n |  | n | 0 |  | 0 | 0 | 0 | 0 | 0 |
| 2011 | 879 | n | y | n |  | p | 0 |  | 0 | 0 | 0 | 0 | 0 |
| 2011 | 880 | n | y | n |  |  | 0 |  | 0 | 0 | 0 | 0 | 0 |
| 2011 | 881 | n | y | n |  |  | 0 |  | 0 | 0 | 0 | 0 | 0 |
| 2011 | 882 | y | y | n |  | n | 0 |  | 0 | 0 | 0 | 0 | 0 |
| 2011 | 883 | n | y | n |  | n | 0 |  | 0 | 0 |  | 0 | 0 |
| 2011 | 884 | y | y | n |  | n | 0 |  | 63 | 0 | 141 | 31 | 0 |
| 2011 | 885 | y | y | n |  | n | 0 |  | 0 | 0 | 110 | 0 | 0 |
| 2011 | 886 | y | n | n |  | n | 0 |  | 15 | 0 | 0 | 106 | 0 |
| 2011 | 887 | y | y | n |  | n | 0 |  | 0 | 0 | 0 | 0 | 0 |
| 2011 | 888 | y | n | n |  | n | 0 |  | 52 | 0 | 74 | 0 | 0 |
| 2011 | 889 | y | n | n |  | n | 0 |  | 38 | 0 | 0 | 0 | 0 |
| 2011 | 890 | n | y | n |  |  | 0 |  | 0 | 0 | 0 | 0 | 0 |
| 2011 | 891 | y | y | n |  |  | 0 |  | 52 | 0 | 0 | 0 | 0 |
| 2011 | 892 | n | n | n |  | n | 0 |  | 0 | 0 | 35 | 0 | 0 |
| 2011 | 893 | y | n | n |  | n | 0 |  | 0 | 0 | 0 | 0 | 0 |
| 2011 | 894 | n | y | n |  | n | 0 |  | 0 | 0 | 0 | 0 | 0 |
| 2011 | 895 | y | n | n |  | n | 0 |  | 20 | 0 | 65 | 0 | 0 |
| 2011 | 896 | n | y | n |  | p | 0 |  | 0 | 0 | 0 | 0 | 0 |
| 2011 | 897 | n | y | n |  | p | 0 |  | 14 | 0 | 0 | 0 | 0 |
| 2011 | 898 | n | y | n |  | n | 0 |  | 0 | 0 | 10 | 21 | 0 |
| 2011 | 899 | y | y | n |  | n | 0 |  | 0 |  | 8 | 30 | 0 |
| 2011 | 900 | y | y | n |  | n | 0 |  | 0 | 0 | 128 | 0 | 0 |
| 2012 | 1 | y | y | n |  | n | 0 | 0 | 180 | 160 | 24 | 0 | 104 |
| 2012 | 2 | n | y | n |  | n | 0 | 0 | 0 | 0 | 0 | 103 | 0 |
| 2012 | 3 | n | y | y | B | p | 0 | 0 | 0 | 0 | 0 | 0 |  |
| 2012 | 4 | y | y | n |  | p | 0 | 0 | 329 | 205 | 0 | 187 | 108 |
| 2012 | 5 | y | y | n |  | n | 0 | 6 | 220 | 235 | 133 | 66 | 90 |
| 2012 | 6 | y | y | n |  | p | 0 | 4 | 274 | 221 | 0 | 87 | 72 |
| 2012 | 7 | n | y | n |  | n | 0 | 0 | 0 | 0 | 201 | 0 | 0 |
| 2012 | 8 | n | y | n |  | n | 0 | 0 | 0 | 0 | 0 | 0 | 0 |
| 2012 | 9 | n | y | n |  | p | 0 | 0 | 19 | 0 | 0 | 0 | 0 |
| 2012 | 10 | n | y | n |  |  | 0 | 0 | 0 | 0 | 0 | 0 | 0 |
| 2012 | 11 | y | y | n |  | n | 0 | 0 | 98 | 6 | 0 | 0 | 0 |
| 2012 | 12 | y | y | y | A | p | 0 | 0 | 203 | 126 | 0 | 41 |  |
| 2012 | 13 | y | y | n |  | n | 0 | 6 | 90 | 54 | 58 | 0 | 0 |
| 2012 | 14 | y | y | n |  | n | 0 | 62 | 288 | 259 | 9 | 26 | 26 |
| 2012 | 15 | n | y | n |  | n | 0 | 0 | 19 | 0 | 0 | 0 | 0 |
| 2012 | 16 | y | y | n |  | n | 0 | 55 | 239 | 198 | 101 | 53 | 72 |
| 2012 | 17 | n | y | n |  | n | 0 | 0 | 0 | 0 | 0 | 0 | 0 |
| 2012 | 18 | y | y | n |  | p | 0 | 80 | 314 | 130 | 57 | 122 | 35 |
| 2012 | 19 | y | y | n |  | n | 0 | 135 | 181 | 147 | 0 | 52 | 0 |
| 2012 | 20 | n | y | n |  | n | 0 | 0 | 0 | 0 | 0 | 0 | 0 |
| 2012 | 21 | y | y | n |  | n | 0 | 0 | 118 | 33 | 60 | 113 | 0 |
| 2012 | 22 | y | y | n |  | n | 0 | 43 | 285 | 179 | 125 | 186 | 0 |
| 2012 | 23 | y | y | n |  | n | 0 | 55 | 295 | 117 | 121 | 118 | 0 |
| 2012 | 24 | n | y | n |  | n | 0 | 0 | 0 | 0 | 0 | 0 | 0 |
| 2012 | 25 | n | y | n |  | p | 0 | 0 | 0 | 0 | 0 | 0 | 0 |
| 2012 | 26 | y | y | n |  | n | 0 | 0 | 3 | 18 | 0 | 0 | 0 |
| 2012 | 27 | y | y | n |  | n | 0 | 14 | 241 | 223 | 94 | 177 | 89 |
| 2012 | 28 | y | y | n |  | p | 0 | 173 | 104 | 205 | 0 | 0 | 0 |
| 2012 | 29 | n | y | n |  | n | 0 | 0 | 0 | 0 | 0 | 0 | 0 |
| 2012 | 30 | n | y | n |  | n | 0 | 0 | 0 | 0 | 0 | 0 | 0 |
| 2012 | 31 | n | y | n |  | n | 0 | 0 | 0 | 0 | 0 | 0 | 0 |
| 2012 | 32 | n | y | n |  | p | 0 | 0 | 0 | 0 | 0 | 0 | 0 |
| 2012 | 33 | y | y | n |  | n | 0 | 39 | 174 | 137 | 63 | 102 | 0 |
| 2012 | 34 | y | y | n |  | p | 0 | 0 | 198 | 127 | 101 | 229 | 161 |
| 2012 | 35 | n | y | n |  | n | 0 | 0 | 0 | 0 | 0 | 0 | 0 |
| 2012 | 36 | y | y | n |  | n | 0 | 0 | 158 | 188 | 78 | 12 | 44 |
| 2012 | 37 | n | y | n |  | p | 0 | 0 | 0 | 0 | 0 | 0 | 0 |
| 2012 | 38 | n | y | n |  | n | 0 | 3 | 0 | 3 | 0 | 1 | 0 |
| 2012 | 39 | y | y | n |  | n | 0 | 0 | 127 | 91 | 88 | 80 | 60 |
| 2012 | 40 | y | y | n |  | n | 0 | 29 | 97 | 88 | 0 | 30 | 0 |
| 2012 | 41 | n | y | n |  | n | 0 | 0 | 0 | 45 | 0 | 8 | 0 |
| 2012 | 42 | n | y | n |  | n | 0 | 0 | 0 | 0 | 0 | 0 | 0 |
| 2012 | 43 | y | y | n |  | n | 0 | 0 | 298 | 328 | 220 | 221 | 157 |
| 2012 | 44 | y | y | n |  | n | 0 | 0 | 65 | 19 | 0 | 0 | 0 |
| 2012 | 45 | y | y | n |  | n | 0 | 0 | 82 | 127 | 52 | 0 | 0 |
| 2012 | 46 | n | y | n |  |  | 0 | 0 | 0 | 0 | 0 | 0 | 0 |
| 2012 | 47 | n | y | n |  | n | 0 | 0 | 0 | 0 | 0 | 0 | 0 |
| 2012 | 48 | y | y | n |  | n | 0 | 0 | 268 | 208 | 21 | 38 | 0 |
| 2012 | 49 | y | y | n |  | n | 0 | 0 | 142 | 164 | 171 | 38 | 0 |
| 2012 | 50 | y | y | n |  | n | 0 | 0 | 240 | 58 | 71 | 117 | 0 |
| 2012 | 51 | n | y | n |  |  | 0 | 39 | 75 | 46 | 44 | 27 | 0 |
| 2012 | 52 | n | y | n |  | n | 0 | 0 | 0 | 0 | 0 | 0 | 0 |
| 2012 | 53 | n | y | n |  | p | 0 | 0 | 118 | 58 | 16 | 4 | 51 |
| 2012 | 54 | n | y | n |  | p | 0 | 0 | 0 | 0 | 0 | 0 | 0 |
| 2012 | 55 | n | y | n |  | p | 0 | 0 | 0 | 0 | 0 | 0 | 0 |
| 2012 | 56 | y | y | n |  | n | 0 | 35 | 228 | 301 | 170 | 79 | 155 |
| 2012 | 57 | y | y | n |  |  | 0 | 65 | 183 | 96 | 44 | 0 | 95 |
| 2012 | 58 | y | y | n |  | n | 0 | 77 | 100 | 0 | 57 | 81 | 0 |
| 2012 | 59 | n | y | n |  | n | 0 | 0 | 0 | 0 | 7 | 0 | 0 |
| 2012 | 60 | n | y | n |  | p | 0 | 0 | 0 | 0 | 0 | 0 | 0 |
| 2012 | 61 | y | y | n |  | n | 0 | 109 | 132 | 125 | 31 | 13 | 0 |
| 2012 | 62 | n | y | n |  | n | 0 | 0 | 0 | 0 | 0 | 0 | 10 |
| 2012 | 63 | n | y | n |  | n | 0 | 0 | 0 | 0 | 0 | 0 | 0 |
| 2012 | 64 | y | y | n |  | n | 0 | 106 | 257 | 179 | 130 | 158 | 168 |
| 2012 | 65 | y | y | n |  | n | 0 | 56 | 31 | 21 | 3 | 28 | 0 |
| 2012 | 66 | y | y | n |  | p | 0 | 0 | 86 | 114 | 74 | 52 | 110 |
| 2012 | 67 | y | y | n |  | n | 0 | 0 | 209 | 261 | 112 | 0 | 21 |
| 2012 | 68 | n | y | n |  | p | 0 | 0 | 0 | 0 | 0 | 0 | 0 |
| 2012 | 69 | n | y | n |  | p | 0 | 0 | 0 | 0 | 0 | 0 | 0 |
| 2012 | 70 | y | y | n |  | n | 0 | 0 | 181 | 44 | 31 | 32 | 59 |
| 2012 | 71 | y | y | y | C |  | 0 | 0 | 189 |  |  |  |  |
| 2012 | 72 | y | y | n |  | n | 0 | 31 | 105 | 69 | 0 | 0 | 37 |
| 2012 | 73 | n | y | n |  | n | 140 | 0 | 0 | 0 | 0 | 38 | 28 |
| 2012 | 74 | n | y | n |  | p | 0 | 0 | 0 | 0 | 0 | 0 | 4 |
| 2012 | 75 | n | y | n |  | p | 0 | 0 | 0 | 0 | 0 | 0 | 0 |
| 2012 | 76 | n | y | n |  | p | 0 | 0 | 0 | 0 | 0 | 0 | 33 |
| 2012 | 77 | y | y | n |  | n | 0 | 60 | 285 | 60 | 174 | 149 | 279 |
| 2012 | 78 | y | y | n |  |  | 0 | 27 | 205 | 27 | 168 | 164 | 225 |
| 2012 | 79 | y | y | n |  | n | 0 | 55 | 247 | 55 | 184 | 126 | 0 |
| 2012 | 80 | y | y | n |  | n | 0 | 27 | 228 | 27 | 84 | 61 | 99 |
| 2012 | 81 | n | y | y | B | p | 0 | 0 | 0 | 0 | 0 |  |  |
| 2012 | 82 | n | y | n |  |  | 0 | 0 | 0 | 0 | 0 | 0 | 91 |
| 2012 | 83 | y | y | n |  | n | 0 | 201 | 242 | 201 | 71 | 0 | 66 |
| 2012 | 84 | n | y | n |  | n | 0 | 11 | 0 | 11 | 0 | 0 | 0 |
| 2012 | 85 | n | y | n |  | p | 0 | 0 | 0 | 0 | 0 | 0 | 8 |
| 2012 | 86 | n | y | n |  | n | 0 | 0 | 0 | 0 | 0 | 0 | 164 |
| 2012 | 87 | y | y | n |  | p | 0 | 78 | 157 | 78 | 103 | 0 | 188 |
| 2012 | 88 | n | y | n |  | p | 0 | 0 | 0 | 0 | 0 | 0 | 39 |
| 2012 | 89 | y | y | n |  | n | 0 | 199 | 195 | 199 | 1 | 71 | 139 |
| 2012 | 90 | n | y | n |  | n | 0 | 0 | 0 | 0 | 0 | 0 | 70 |
| 2012 | 91 | n | n | n |  | n | 0 | 0 | 0 | 0 | 0 | 0 | 56 |
| 2012 | 92 | y | y | n |  | n | 0 | 11 | 124 | 11 | 77 | 59 | 142 |
| 2012 | 93 | n | y | n |  | n | 0 | 17 | 0 | 17 | 0 | 0 | 64 |
| 2012 | 94 | y | y | n |  |  | 0 | 66 | 155 | 66 | 43 | 28 | 119 |
| 2012 | 95 | n | y | n |  | n | 0 | 0 | 0 | 0 | 0 | 0 | 132 |
| 2012 | 96 | n | y | n |  | n | 0 | 0 | 0 | 0 | 0 | 0 | 148 |
| 2012 | 97 | n | y | n |  | p | 0 | 46 | 0 | 32 | 0 | 26 | 0 |
| 2012 | 98 | y | y | n |  | n | 0 | 18 | 241 | 270 | 131 | 140 | 126 |
| 2012 | 99 | n | y | n |  | p | 0 | 9 | 11 | 6 | 0 | 0 | 0 |
| 2012 | 100 | y | y | n |  | n | 0 | 0 | 197 | 110 | 22 | 107 | 54 |

Table 3. Virus neutralising assay data.

| **Trial** | **ID** | **Vacc** | **Clinical** | **Died** | **Histopath** | **Inf. Status** | **VNA-Pl 0** | **VNA-Pl 2** | **VNA-NS 0** | **VNA-NS 2** |
| --- | --- | --- | --- | --- | --- | --- | --- | --- | --- | --- |
| 2011 | 801 | y | y | n |  | n |  |  |  | 0 |
| 2011 | 802 | y | y | n |  | n |  |  |  | 0 |
| 2011 | 803 | n | y | n |  | n |  |  |  |  |
| 2011 | 804 | n | y | n |  | n |  |  |  |  |
| 2011 | 805 | n | y | n |  | n | 0 | 6 | 0 | 0 |
| 2011 | 806 | y | n | n |  | n |  |  |  | 0 |
| 2011 | 807 | n | y | n |  | n |  |  |  |  |
| 2011 | 808 | n | y | n |  | n |  |  |  |  |
| 2011 | 809 | y | y | n |  | n | 0 | 128 |  | 106 |
| 2011 | 810 | n | y | n |  | n |  |  |  |  |
| 2011 | 811 | y | y | n |  | n |  |  |  |  |
| 2011 | 812 | n | y | n |  | p | 0 | 8 | 0 | 0 |
| 2011 | 813 | y | y | y | A | p |  |  |  | 0 |
| 2011 | 814 | n | y | n |  | p |  |  |  | 0 |
| 2011 | 815 | n | y | n |  | p |  |  |  | 0 |
| 2011 | 816 | y | y | n |  | n |  |  |  | 64 |
| 2011 | 817 | n | y | n |  | n |  |  |  |  |
| 2011 | 818 | n | y | n |  | p |  |  |  |  |
| 2011 | 819 | y | y | n |  | p |  |  |  | 0 |
| 2011 | 820 | y | y | n |  | n | 0 |  |  | 64 |
| 2011 | 821 | y | y | n |  | n |  |  |  | 8 |
| 2011 | 822 | y | n | n |  | n |  |  |  | 0 |
| 2011 | 823 | n | y | n |  | n |  |  |  |  |
| 2011 | 824 | y | y | y | A | p |  |  |  | 0 |
| 2011 | 825 | n | n | n |  | n |  |  |  |  |
| 2011 | 826 | y | y | n |  | n | 0 | 90 | 0 | 68 |
| 2011 | 827 | n | y | n |  | n |  |  |  |  |
| 2011 | 828 | y | y | n |  | p |  |  |  | 3 |
| 2011 | 829 | y | y | n |  | n |  |  |  | 0 |
| 2011 | 830 | n | y | n |  | p |  |  |  |  |
| 2011 | 831 | y | y | n |  | n |  |  |  | 11 |
| 2011 | 832 | n | n | n |  | p |  |  |  |  |
| 2011 | 833 | n | y | n |  | n |  |  |  |  |
| 2011 | 834 | y | y | n |  | p |  |  |  | 6 |
| 2011 | 835 | n | n | n |  | p |  |  |  |  |
| 2011 | 836 | n | y | n |  | p |  |  |  |  |
| 2011 | 837 | n | y | n |  | p |  |  |  |  |
| 2011 | 838 | n | y | n |  | p |  |  |  |  |
| 2011 | 839 | y | y | n |  | p |  |  |  | 0 |
| 2011 | 840 | n | y | n |  | p |  |  |  |  |
| 2011 | 841 | y | y | n |  | n |  |  |  | 0 |
| 2011 | 842 | y | y | n |  | p |  |  |  | 0 |
| 2011 | 843 | n | y | n |  | p |  |  |  |  |
| 2011 | 844 | y | y | n |  | n |  |  |  | 3 |
| 2011 | 845 | y | n | n |  | n |  |  |  |  |
| 2011 | 846 | y | y | n |  | n | 0 | 8 | 0 | 55 |
| 2011 | 847 | y | y | n |  | p | 0 | 6 | 0 | 14 |
| 2011 | 848 | n | y | n |  | p |  |  |  |  |
| 2011 | 849 | y | y | n |  | n |  |  |  | 6 |
| 2011 | 850 | y | y | n |  | n |  |  |  | 6 |
| 2011 | 851 | n | y | n |  | p |  |  |  |  |
| 2011 | 852 | y | n | n |  | n |  |  |  | 22 |
| 2011 | 853 | y | y | n |  | n | 0 | 32 |  | 45 |
| 2011 | 854 | n | y | n |  | p |  |  |  |  |
| 2011 | 855 | y | y | n |  | p |  |  |  | 0 |
| 2011 | 856 | n | y | n |  | n |  |  |  |  |
| 2011 | 857 | n | y | y | A | p | 0 |  | 0 |  |
| 2011 | 858 | n | y | n |  | n |  |  |  |  |
| 2011 | 859 | n | y | n |  | p |  |  |  |  |
| 2011 | 860 | y | y | n |  | n |  |  |  | 11 |
| 2011 | 861 | y | y | n |  | n |  |  |  | 64 |
| 2011 | 862 | n | y | n |  | n |  |  |  |  |
| 2011 | 863 | n | y | n |  | n |  |  |  |  |
| 2011 | 864 | y | y | n |  | n |  |  |  | 45 |
| 2011 | 865 | n | y | n |  | p |  |  |  | 0 |
| 2011 | 866 | n | y | n |  | n |  |  |  |  |
| 2011 | 867 | y | y | n |  | p |  |  |  | 0 |
| 2011 | 868 | n | y | n |  | n | 0 | 3 | 0 | 0 |
| 2011 | 869 | n | y | y | B | p |  |  |  |  |
| 2011 | 870 | n | y | n |  |  |  |  |  |  |
| 2011 | 871 | y | n | n |  | n |  |  |  | 45 |
| 2011 | 872 | n | y | n |  | n |  |  |  |  |
| 2011 | 873 | n | n | n |  | n |  |  |  | 0 |
| 2011 | 874 | y | n | n |  | n |  |  |  | 32 |
| 2011 | 875 | y | y | n |  | n | 0 | 180 | 0 | 55 |
| 2011 | 876 | y | y | n |  |  | 0 | 22 | 0 | 45 |
| 2011 | 877 | y | y | n |  | n |  |  |  | 0 |
| 2011 | 878 | n | y | n |  | n |  |  |  | 0 |
| 2011 | 879 | n | y | n |  | p |  |  |  | 0 |
| 2011 | 880 | n | y | n |  |  |  |  |  | 0 |
| 2011 | 881 | n | y | n |  |  |  |  |  | 0 |
| 2011 | 882 | y | y | n |  | n |  |  |  | 0 |
| 2011 | 883 | n | y | n |  | n |  |  |  |  |
| 2011 | 884 | y | y | n |  | n | 0 | 90 | 0 | 34 |
| 2011 | 885 | y | y | n |  | n |  |  |  | 0 |
| 2011 | 886 | y | n | n |  | n |  |  |  | 0 |
| 2011 | 887 | y | y | n |  | n |  |  |  | 6 |
| 2011 | 888 | y | n | n |  | n | 3 |  | 0 | 64 |
| 2011 | 889 | y | n | n |  | n |  |  |  | 6 |
| 2011 | 890 | n | y | n |  |  |  |  |  |  |
| 2011 | 891 | y | y | n |  |  | 0 |  | 0 | 11 |
| 2011 | 892 | n | n | n |  | n |  |  |  |  |
| 2011 | 893 | y | n | n |  | n |  |  |  | 0 |
| 2011 | 894 | n | y | n |  | n |  |  |  |  |
| 2011 | 895 | y | n | n |  | n |  |  |  | 0 |
| 2011 | 896 | n | y | n |  | p |  |  |  |  |
| 2011 | 897 | n | y | n |  | p |  |  |  |  |
| 2011 | 898 | n | y | n |  | n |  |  |  |  |
| 2011 | 899 | y | y | n |  | n |  |  |  | 4 |
| 2011 | 900 | y | y | n |  | n |  |  |  | 0 |
| 2012 | 1 | y | y | n |  | n |  |  |  | 11 |
| 2012 | 2 | n | y | n |  | n |  |  |  |  |
| 2012 | 3 | n | y | y | B | p |  |  |  |  |
| 2012 | 4 | y | y | n |  | p | 0 | 180 | 0 | 109 |
| 2012 | 5 | y | y | n |  | n |  |  |  | 64 |
| 2012 | 6 | y | y | n |  | p | 0 | 256 | 0 | 51 |
| 2012 | 7 | n | y | n |  | n |  |  |  |  |
| 2012 | 8 | n | y | n |  | n |  |  |  |  |
| 2012 | 9 | n | y | n |  | p |  |  | 0 | 0 |
| 2012 | 10 | n | y | n |  |  |  |  |  |  |
| 2012 | 11 | y | y | n |  | n |  |  |  | 22 |
| 2012 | 12 | y | y | y | A | p |  |  |  | 16 |
| 2012 | 13 | y | y | n |  | n |  |  |  | 45 |
| 2012 | 14 | y | y | n |  | n | 0 | 1024 | 0 | 173 |
| 2012 | 15 | n | y | n |  | n |  |  | 0 | 0 |
| 2012 | 16 | y | y | n |  | n |  |  |  | 90 |
| 2012 | 17 | n | y | n |  | n |  |  |  |  |
| 2012 | 18 | y | y | n |  | p | 0 | 128 | 0 | 101 |
| 2012 | 19 | y | y | n |  | n |  |  |  | 128 |
| 2012 | 20 | n | y | n |  | n |  |  |  |  |
| 2012 | 21 | y | y | n |  | n |  |  |  | 4 |
| 2012 | 22 | y | y | n |  | n | 0 | 90 | 0 | 55 |
| 2012 | 23 | y | y | n |  | n | 0 | 128 | 0 | 11 |
| 2012 | 24 | n | y | n |  | n |  |  |  |  |
| 2012 | 25 | n | y | n |  | p |  |  |  |  |
| 2012 | 26 | y | y | n |  | n |  |  |  |  |
| 2012 | 27 | y | y | n |  | n | 0 | 90 | 0 | 22 |
| 2012 | 28 | y | y | n |  | p |  |  |  | 45 |
| 2012 | 29 | n | y | n |  | n |  |  |  |  |
| 2012 | 30 | n | y | n |  | n |  |  |  |  |
| 2012 | 31 | n | y | n |  | n |  |  |  |  |
| 2012 | 32 | n | y | n |  | p |  |  |  |  |
| 2012 | 33 | y | y | n |  | n |  |  |  | 128 |
| 2012 | 34 | y | y | n |  | p |  |  |  | 0 |
| 2012 | 35 | n | y | n |  | n |  |  |  |  |
| 2012 | 36 | y | y | n |  | n |  |  |  | 22 |
| 2012 | 37 | n | y | n |  | p |  |  |  |  |
| 2012 | 38 | n | y | n |  | n |  |  |  |  |
| 2012 | 39 | y | y | n |  | n |  |  |  | 22 |
| 2012 | 40 | y | y | n |  | n |  |  |  | 0 |
| 2012 | 41 | n | y | n |  | n |  |  |  |  |
| 2012 | 42 | n | y | n |  | n |  |  |  |  |
| 2012 | 43 | y | y | n |  | n | 0 | 64 | 0 | 113 |
| 2012 | 44 | y | y | n |  | n |  |  |  | 8 |
| 2012 | 45 | y | y | n |  | n |  |  |  | 22 |
| 2012 | 46 | n | y | n |  |  |  |  |  |  |
| 2012 | 47 | n | y | n |  | n |  |  |  |  |
| 2012 | 48 | y | y | n |  | n | 0 | 180 | 0 | 53 |
| 2012 | 49 | y | y | n |  | n |  |  |  | 6 |
| 2012 | 50 | y | y | n |  | n |  |  |  | 11 |
| 2012 | 51 | n | y | n |  |  |  |  | 0 | 0 |
| 2012 | 52 | n | y | n |  | n |  |  |  | 0 |
| 2012 | 53 | n | y | n |  | p |  |  | 0 | 0 |
| 2012 | 54 | n | y | n |  | p |  |  |  |  |
| 2012 | 55 | n | y | n |  | p | 0 | 0 |  | 0 |
| 2012 | 56 | y | y | n |  | n |  |  |  | 64 |
| 2012 | 57 | y | y | n |  |  |  |  |  | 128 |
| 2012 | 58 | y | y | n |  | n |  |  |  |  |
| 2012 | 59 | n | y | n |  | n |  |  |  |  |
| 2012 | 60 | n | y | n |  | p | 0 | 0 |  | 0 |
| 2012 | 61 | y | y | n |  | n |  |  |  |  |
| 2012 | 62 | n | y | n |  | n |  |  |  |  |
| 2012 | 63 | n | y | n |  | n | 0 | 0 |  | 0 |
| 2012 | 64 | y | y | n |  | n | 0 | 180 | 0 | 34 |
| 2012 | 65 | y | y | n |  | n |  |  |  | 45 |
| 2012 | 66 | y | y | n |  | p |  |  |  | 22 |
| 2012 | 67 | y | y | n |  | n |  |  |  |  |
| 2012 | 68 | n | y | n |  | p |  |  |  |  |
| 2012 | 69 | n | y | n |  | p | 0 | 0 |  | 0 |
| 2012 | 70 | y | y | n |  | n |  |  |  | 90 |
| 2012 | 71 | y | y | y | C |  |  |  |  | 45 |
| 2012 | 72 | y | y | n |  | n |  |  |  | 22 |
| 2012 | 73 | n | y | n |  | n |  |  |  |  |
| 2012 | 74 | n | y | n |  | p |  |  |  |  |
| 2012 | 75 | n | y | n |  | p |  |  |  |  |
| 2012 | 76 | n | y | n |  | p |  |  |  |  |
| 2012 | 77 | y | y | n |  | n | 0 | 360 | 0 | 16 |
| 2012 | 78 | y | y | n |  |  |  |  |  | 128 |
| 2012 | 79 | y | y | n |  | n | 0 | 720 | 0 | 16 |
| 2012 | 80 | y | y | n |  | n |  |  |  | 45 |
| 2012 | 81 | n | y | y | B | p |  |  |  |  |
| 2012 | 82 | n | y | n |  |  |  |  |  |  |
| 2012 | 83 | y | y | n |  | n | 0 | 180 | 0 | 27 |
| 2012 | 84 | n | y | n |  | n |  |  |  |  |
| 2012 | 85 | n | y | n |  | p |  |  |  |  |
| 2012 | 86 | n | y | n |  | n |  |  |  |  |
| 2012 | 87 | y | y | n |  | p |  |  |  | 128 |
| 2012 | 88 | n | y | n |  | p |  |  |  |  |
| 2012 | 89 | y | y | n |  | n |  |  |  | 256 |
| 2012 | 90 | n | y | n |  | n |  |  |  |  |
| 2012 | 91 | n | n | n |  | n |  |  |  |  |
| 2012 | 92 | y | y | n |  | n |  |  |  | 22 |
| 2012 | 93 | n | y | n |  | n |  |  |  | 0 |
| 2012 | 94 | y | y | n |  |  |  |  |  | 180 |
| 2012 | 95 | n | y | n |  | n |  |  |  |  |
| 2012 | 96 | n | y | n |  | n |  |  |  |  |
| 2012 | 97 | n | y | n |  | p |  |  |  |  |
| 2012 | 98 | y | y | n |  | n |  |  |  | 22 |
| 2012 | 99 | n | y | n |  | p |  |  |  |  |
| 2012 | 100 | y | y | n |  | n |  |  |  | 45 |
