# Supplementary material for: A field vaccine trial in Tanzania demonstrates partial protection against malignant catarrhal fever in cattle
Source: Vaccine. 2016 Feb 3;34(6):831–8. doi: 10.1016/j.vaccine.2015.12.009 (PMC4742522; doi:10.1016/j.vaccine.2015.12.009)
Supplement: Supplementary Data 2 — How the wildebeest calf Contact Index was measured and calculated is described, plus a table is presented that shows the wildebeest calf contact data for each five-day period of the 2011 trial. [file mmc2.docx]

**Supplementary Data 2: Wildebeest calf contact data**

In each trial the challenge phase began in mid-February and lasted until the end of May. During this period, cattle were grazed as a single herd as close as possible to wildebeest and their calves. This pattern is in contrast to traditionally managed herds, which are moved away from wildebeest at this time of year. To estimate the daily intensity of challenge, the Contact Index, which reflects the intensity of contact between the trial cattle and wildebeest calves, was calculated during the 2011 trial using the equation:

$$Daily contact index=\alpha\times\beta\times\gamma$$

Where ($\alpha)$is the duration (minutes) that the trial cattle spent grazing within 200 metres of wildebeest calves, ($\beta$) is the number of wildebeest calves present during these encounters and ($\gamma$) is an ordinal value that represents the minimum recorded distance between the calves and trial cattle during the period (zero to 50 metres = 3, 51 to 100 metres = 2, and > 100 metres = 1 (measured by Bushnell® golf range-finder; Bushnell-UK, Chessington, UK).

The table below shows wildebeest calf contact data for each five-day period of the 2011 trial.

| Period (days) | Duration (mins) (α) |  | Wildebeest calves (β) | Distance (m) | Ordinal value (γ) | Contact index |
| --- | --- | --- | --- | --- | --- | --- |
| 1-5 | 108 |  | 12 | 82 | 2 | 2,584 |
| 6-10 | 85 |  | 5 | 31 | 3 | 1,268 |
| 11-15 | 282 |  | 8 | 57 | 2 | 4,512 |
| 16-20 | 190 |  | 36 | 70 | 2 | 13,739 |
| 21-25 | 186 |  | 85 | 121 | 1 | 15,810 |
| 26-30 | 166 |  | 142 | 287 | 1 | 23,564 |
| 31-35 | 175 |  | 53 | 197 | 1 | 9,262 |
| 46-50 | 177 |  | 112 | 77 | 2 | 39,360 |
| 51-55 | 331 |  | 19 | 84 | 2 | 12,578 |
| 56-60 | 258 |  | 20 | 110 | 3 | 15,480 |
| 61-65 | 249 |  | 25 | 58 | 2 | 12,177 |
| 66-70 | 134 |  | 33 | 85 | 2 | 8,844 |
| 71-75 | 331 |  | 25 | 91 | 2 | 16,525 |
| 76-80 | 172 |  | 60 | 70 | 2 | 20,640 |
| 81-85 | 324 |  | 42 | 44 | 3 | 40,824 |
| 86-90 | 320 |  | 22 | 98 | 2 | 14,058 |
| 91-95 | 193 |  | 36 | 83 | 2 | 13,896 |
| 96-100 | 211 |  | 25 | 68 | 2 | 10,339 |
| 101-105 | 111 |  | 9 | 116 | 1 | 999 |
| 106-110 | 0 |  | 0 | NA | 0 | 0 |
