# Supplementary material for: A field vaccine trial in Tanzania demonstrates partial protection against malignant catarrhal fever in cattle
Source: Vaccine. 2016 Feb 3;34(6):831–8. doi: 10.1016/j.vaccine.2015.12.009 (PMC4742522; doi:10.1016/j.vaccine.2015.12.009)
Supplement: Supplementary Data 3 — The selection of plasma and nasal secretion samples for virus neutralizing antibody (VNA) assay is described. [file mmc3.docx]

**Supplementary data 3: Selection of samples for virus neutralizing antibody (VNA) assay**

| Sample type | Trial | Group | Time point | Number of cattle assayed |
| --- | --- | --- | --- | --- |
| Plasma | 2011 | Unvaccinated | Day zero | 3 |
| Nasal secretion | 2011 | Unvaccinated | Day zero | 3 |
| Plasma | 2011 | Unvaccinated | Two-month | 2 |
| Nasal secretion | 2011 | Unvaccinated | Two-month | 11 |
| Plasma | 2011 | Vaccinated | Day zero | 8 |
| Nasal secretion | 2011 | Vaccinated | Day zero | 8 |
| Plasma | 2011 | Vaccinated | Two-month | 7 |
| Nasal secretion | 2011 | Vaccinated | Two-month | 48 |
| Plasma | 2012 | Unvaccinated | Day zero | 4 |
| Nasal secretion | 2012 | Unvaccinated | Day zero | 4 |
| Plasma | 2012 | Unvaccinated | Two-month | 4 |
| Nasal secretion | 2012 | Unvaccinated | Two-month | 10 |
| Plasma | 2012 | Vaccinated | Day zero | 13 |
| Nasal secretion | 2012 | Vaccinated | Day zero | 13 |
| Plasma | 2012 | Vaccinated | Two-month | 13 |
| Nasal secretion | 2012 | Vaccinated | Two-month | 46 |

The VNA analyses were conducted on a sample of the trial cattle at the time of primary vaccination and at the two-month time point, as detailed above. The vaccinated cattle selected for VNA analysis were chosen at random, while all of the unvaccinated cattle with ELISA titres >20 were used.
