# Supplementary material for: A field vaccine trial in Tanzania demonstrates partial protection against malignant catarrhal fever in cattle
Source: Vaccine. 2016 Feb 3;34(6):831–8. doi: 10.1016/j.vaccine.2015.12.009 (PMC4742522; doi:10.1016/j.vaccine.2015.12.009)
Supplement: Supplementary Data 5 — The method and data used for the vaccine efficacy calculation are described. [file mmc5.docx]

**Supplementary Data 5: MCF vaccine efficacy**

Vaccine efficacy ($VE)$ is equivalent to the percentage reduction in the incidence of a specific outcome (or ‘case’) that is attributable to the vaccine and is calculated using the notation and formula shown in Supplementary Data 5 Table A [26]. Vaccine efficacy confidence intervals were calculated using established formulae [27].

**Table A: A vaccine efficacy calculation 2 x 2 table**:

| Metric Group Case Non-case Total Risk Efficacy | | |
| --- | --- | --- |
| ‘Case’ | Vaccinated $a$ $b$ $a+ b$ $ARv = \frac{a}{(a+b)}$  Unvaccinated $c$ $d$ $c+ d$ $ARu = \frac{c}{(c+d)}$ | $VE = 1- \frac{ARv}{ARu} \times100$ |

*a* & c are the number of ‘cases’ in vaccinated and unvaccinated cattle respectively; *b* & *d* are the number of ‘non-cases’ in vaccinated and unvaccinated cattle respectively; $ARv$ & $ARu$are the attack rates in vaccinated and unvaccinated cattle respectively; the vaccine efficacy ($VE)$ at reducing the incidence of ‘cases’ is equivalent to the percentage reduction in ‘cases’ that was attributable to the vaccine [26].

To determine the $VE$ for reducing AlHV-1 infection, PCR data from both the 2011 and 2012 trials were used to populate the Supplementary Data 5 Table B according to the method described above and the $VE$ at reducing AlHV-1 infection was estimated.

**Table B:** **Calculating the efficacy of the MCF vaccine at preventing AlHV-1 infection**

| Metric Group Case Non-case Total Risk Efficacy | | |
| --- | --- | --- |
| AlHV-1 infection | Vaccinated 19 76 95 0.20  Unvaccinated $41$ 51 92 0.45 | $VE$ = 56%  (CI: 17 to 97%) |
